# Regional climate contributes more than geographic distance to beta diversity of copepods (Crustacea Copepoda) between caves of Italy

**DOI:** 10.1038/s41598-023-48440-7

**Published:** 2023-12-01

**Authors:** Emma Galmarini, Ilaria Vaccarelli, Barbara Fiasca, Mattia Di Cicco, Mario Parise, Isabella Serena Liso, Leonardo Piccini, Diana Maria Paola Galassi, Francesco Cerasoli

**Affiliations:** 1 Department of Life, Health and Environmental Sciences, University of L’Aquila, L’Aquila, Italy; 2 University Institute of Higher Studies in Pavia, Pavia, Italy; 3 Department of Earth and Environmental Sciences, University Aldo Moro, Bari, Italy; 4 Department of Earth Science, University of Florence, Florence, Italy

**Keywords:** Zoology, Ecology, Biodiversity, Biogeography, Community ecology, Ecological modelling, Freshwater ecology

## Abstract

Despite the study of subterranean biodiversity facing harsh sampling and mapping challenges, the huge diversity of taxa, ecological adaptations and evolutionary trajectories in subterranean environments is gaining increasing attention. Yet, the spatial and environmental factors driving the composition of groundwater communities are still poorly understood. To partially fill this knowledge gap, we collected copepod crustaceans from 12 caves along the Italian peninsula between 2019 and 2022, sampling each cave twice. The resulting presence-absence data were analysed to assess: (i) between-cave taxonomic beta diversity, also partitioning between turnover and nestedness-resultant dissimilarity; (ii) the relative weight of geographic distance and climatic differences in shaping observed beta diversity. Seventy-one species of copepods were collected overall. Pairwise beta diversity was high for most pairs of caves, with turnover being the major component. Geographic distance-decay models partially explained total beta diversity and turnover patterns. However, in Generalized Dissimilarity Models (GDM), including surface climatic conditions as predictors, the contribution of seasonal temperature averages was generally higher than that of geographic distance. Further, the explanatory and predictive performance of the GDMs notably increased, along with temperature contribution, when widening the spatial extent from which climate data were gathered. Our results confirmed a high spatial turnover in groundwater copepods’ assemblages and strengthened the link between regional climate and subterranean biodiversity.

## Introduction

Despite the subterranean environment harbours a diverse array of living forms, including microorganisms, invertebrates, and vertebrates^1^, its biodiversity has received limited attention in terms of biomonitoring and conservation efforts^2,3^. The exploration of subterranean biodiversity faces a significant challenge called the Racovitzan impediment^4^. This concept encapsulates a set of inherent difficulties encountered in studying and understanding subterranean ecosystems and their dwellers, mostly related to the limited accessibility and the peculiar abiotic (e.g., total darkness, low oxygenation) and biotic (e.g., nutrient scarcity) conditions of most subterranean environments^5^. In recent years, the scientific community has strongly reaffirmed the urgent need to overcome these challenges to advance our understanding of subterranean biodiversity and develop effective conservation strategies^3,6^. Given the aforementioned access and sampling challenges, the picture gleaned from studying subterranean environments is unavoidably limited. In fact, many taxa thriving beneath the surface have yet to be described, while unexpected findings of certain taxa in new areas often defy biogeographic expectations. Alongside the Racovitzan impediment, these taxonomic (i.e., Linnean) and biogeographic (i.e., Wallacean) shortfalls hinder the effectiveness of conservation programs aimed at preserving subterranean biodiversity^2,7^. However, recent advances in speleological techniques and sampling methods have propelled our understanding of subterranean biodiversity forward^8–17^. The growing body of research targeting subterranean environments has revealed remarkable examples of convergent evolution and surprising biological radiations^18,19^, thereby reaffirming that subterranean organisms offer crucial insights into widely debated topics such as the time and mode of speciation^20^.

Accessible caves represent only a fraction of the vast subterranean realm, characterised by macro- and microfractures, conduits, streams and lakes, and large chambers being intermittently or permanently filled with groundwater. Cave systems are particularly extensive and articulated in karst regions, where the hydrologic cycle plays a pivotal role in the formation and functioning of the groundwater environments^21^. As meteoric water infiltrates fractured rocks (e.g., carbonate rocks), it contributes to the development of two distinct yet usually connected zones: the unsaturated and the saturated karst. While the former is characterised by intermittent groundwater availability, the latter is permanently flooded. Both these groundwater “black boxes” host a remarkable variety of habitats supporting diverse biological communities^22,23^. Surprisingly, the unsaturated portions of a karst system, comprising temporary pools, trickles and locally waterfilled fractures in the bedrock, often exhibit higher species richness than the saturated karst within the same system^9^. Furthermore, species found in the unsaturated karst are rarely the same as those collected from the saturated karst, primarily due to differences in habitat availability for obligate groundwater dwellers (i.e., stygobites). Alongside stygobites, aquatic species usually thriving in surface waters (i.e., non-stygobites) are commonly found in both the unsaturated and saturated portions of karst groundwater. Despite their primary habitats being surface waters (e.g., lakes, streams, ponds), they occasionally or accidentally enter groundwater via the connections between the surface and the underground.

Karst aquifers offer valuable ecosystem services, such as provisioning of freshwater for human consumption, irrigation, and industrial use^24^. However, these aquifers are highly sensitive to direct or indirect human impacts^2,25^, including contamination, overexploitation, and climate change^26,27^. Moreover, karst groundwater hosts rare and narrow-range endemic species that are particularly vulnerable to extinction^28–30^. Biological surveys conducted over the past 30 years have provided insights into the large-scale factors influencing the composition of biological assemblages in karst areas, including evolutionary history, habitat heterogeneity and environmental gradients^14,30–36^. Nevertheless, only a few studies^33,36^ have thus far evaluated the relative role of geographic distance and environmental gradients on biotic dissimilarity (i.e., beta diversity) of groundwater crustacean assemblages, explicitly partitioning between spatial turnover and nestedness-resultant dissimilarity^37^. Partitioning beta diversity into these two components and exploring its geographic, historical, and environmental correlates has proven to be crucial for understanding biogeographic processes^36,38,39^ and outlining conservation priorities^40,41^.

In this study, we analysed copepod (Crustacea Copepoda) assemblages from 12 caves spanning seven macro-areas along the Italian peninsula, from the northeastern karst (Friuli-Venezia Giulia) to the southern gypsum area (Calabria) and limestone systems (Apulia). By sampling caves from different macro-areas, we aimed to obtain a representative sample of the copepod diversity across the Italian karst and to investigate the associated ecological dynamics. We selected the Crustacea Copepoda as the target group because they were the most abundant and species-rich taxon in all the sampled caves. With the hypothesis that both spatial isolation and regional climate may have shaped the composition of biotic communities in cave waters, we conducted this research work with the following objectives: (i) to characterise the copepod assemblages of the examined caves in terms of species richness (i.e., alpha diversity); (ii) to quantify between-caves beta diversity; (iii) to evaluate the relative contributions of geographic distance and surface climatic conditions to the observed patterns of beta diversity.

In a broader context, our results contribute to pushing forward the current knowledge about how surface climatic conditions affect faunal diversity in cave waters, a topic of primary importance in the context of the ongoing climate crisis and associated biodiversity loss. Further, by taking advantage of climate data from an online repository to fit state-of-the-art dissimilarity models on presence-absence data of groundwater fauna, our analytical workflow may represent a “template” for other subterranean biologists in exploiting the increasing amount of publicly available and spatially explicit digital environmental data to disentangle the drivers shaping subterranean biodiversity.

## Material and methods

### Study sites

**Figure 1:**
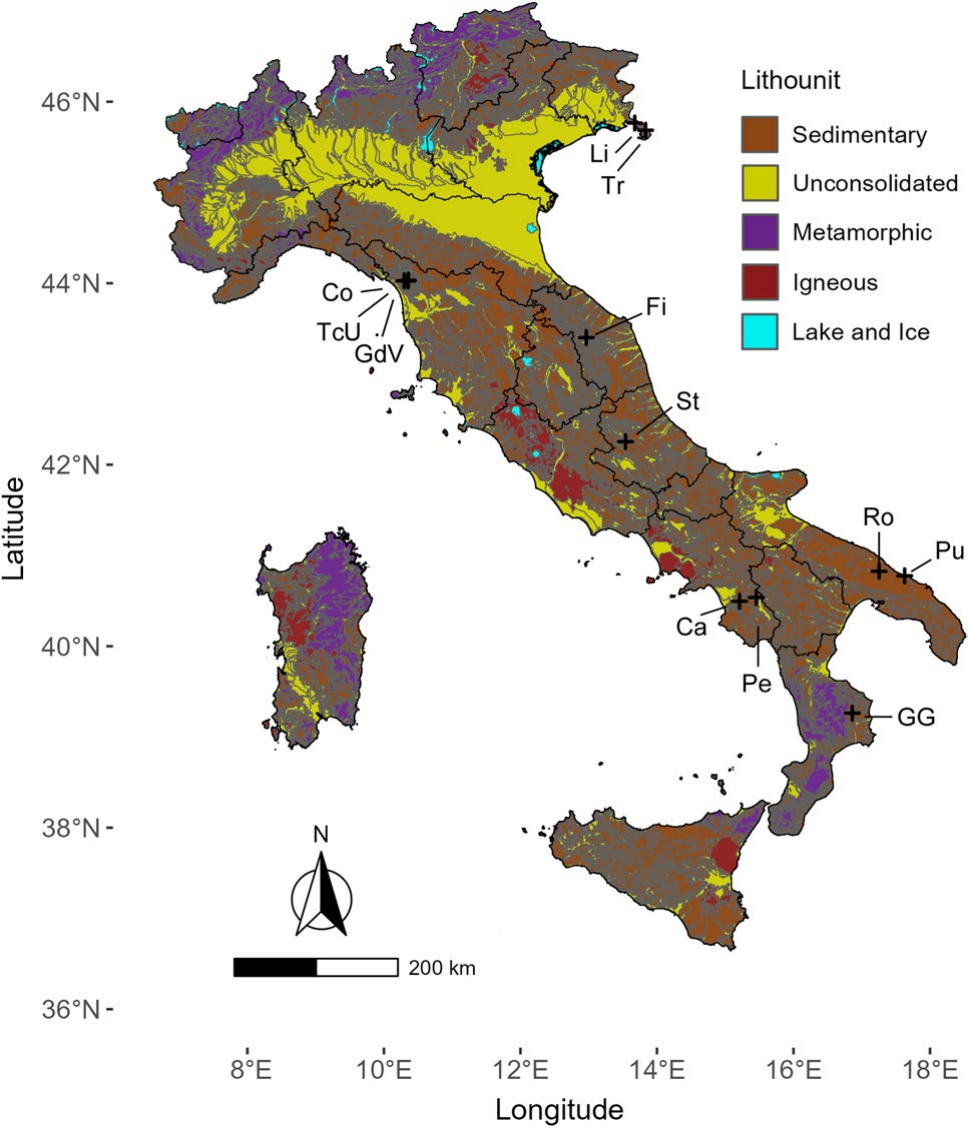
Map of Italian lithounits (based on the Italian lithological map retrieved from the Italian National Geoportal, http://www.pcn.minambiente.it/mattm/en/), with Italian administrative borders (black contour lines), the twelve sampled caves (black crosses) and their corresponding codes (see Table 1).

We selected twelve caves in diverse karst areas across the Italian peninsula, spanning multiple latitudinal bands (Fig. 1), and we sampled each cave twice between 2019 and 2022.

The investigated caves (Table 1) span seven macro-areas with distinct hydrogeologic characteristics: (i) Trieste Karst of the Eastern Alps (Northeastern Italy), which comprises the Abisso di Trebiciano (coded as Tr, see Table 1) and Grotta Antonio Federico Lindner (Li) caves; (ii) the Apuan Alps in the Northern Apennines (Northwestern Italy), encompassing the caves named Antro del Corchia (Co), Tana che Urla (TcU), and Grotta del Vento (GdV); (iii) the Frasassi Gorge area in the Central Apennines (Central Italy), including Grotta del Fiume (Fi); (iv) the karst area of inland Abruzzo in the Central Apennines (Central Italy), hosting Grotte di Stiffe (St); (v) the Alta Murgia and Bassa Murgia subregions of Apulia (Southern Italy), respectively hosting Grave Rotolo (Ro) and Grotta Puntore (Pu); (vi) the Alburni Massif in the Southern Apennines (Southern Italy), with Grotte di Castelcivita (Ca) and Grotte di Pertosa-Auletta (Pe); (vii) the evaporitic rock formations of the Southern Apennines (Southern Italy), with the Grave Grubbo (GG) cave.

**Table 1:** For each sampled cave, the following data are reported: extended and abbreviated (“Cave code”) denomination, coordinates (EPSG 4326: WGS84), elevation (at cave entrance, as derived from a 30 m-resolution DEM), lithological composition, horizontal and vertical extents.

Cave	Cave code	Longitude	Latitude	Elevation (m a.s.l.)	Lithology	Length (m)	Depth (m)
Abisso di Trebiciano	Tr	13.833	45.685	353	Limestone	1198	329
Grotta Lindner	Li	13.675	45.765	179	Limestone	825	117
Antro del Corchia	Co	10.296	44.027	799	Marble and dolomite	74,000	1185
Grotta del Vento	GdV	10.358	44.033	646	Dolomite and carbonate breccias	4600	162
Tana che Urla	TcU	10.349	44.024	655	Dolomite and carbonate breccias	400	48
Grotta del Fiume	Fi	12.965	43.401	257	Limestone	7000	250
Grotte di Stiffe	St	13.540	42.254	803	Limestone	2300	190
Grave Rotolo	Ro	17.254	40.825	298	Limestone	1412	324
Grotta Puntore	Pu	17.628	40.776	28	Limestone	20	13
Grotte di Castelcivita	Ca	15.209	40.495	101	Limestone	5400	33
Grotte di Pertosa-Auletta	Pe	15.455	40.537	244	Limestone	3300	46
Grave Grubbo	GG	16.861	39.262	325	Evaporitic sediments	1926	59

Abisso di Trebiciano and Grotta Antonio Federico Lindner caves are located along the underground course of the Timavo River^42^, the main contributor to the recharge of the Trieste Karst aquifer. During flood periods, the water rises several meters inside the two caves, so that their deepest portions become totally submerged.

Antro del Corchia cave is located on the southwestern slopes of the Apuan Alps and is part of one of Italy’s largest karst systems^43^. Due to its huge overall length (dozens of kilometers) and depth (> 1000 m), and the consequent difficulties in displacing the sampling equipment, in this cave we focused the sampling on the habitats located close to the touristic path, which included an underground stream, some small lakes, pools, and dripping pools. On the southeastern side of the Apuan Alps are Grotta del Vento and Tana che Urla caves. Most of the former cave is characterized by unsaturated habitats, with an underground stream flowing only in its deepest part. Contrarily, Tana che Urla is crossed throughout its development by a perennial stream, without a well-developed unsaturated zone.

Grotta del Fiume has a sub-horizontal development, spanning 7 km and hosting several lakes and dripping pools, with both the saturated and unsaturated parts of the cavity being well-developed.

Grotte di Stiffe cave is traversed throughout by a perennial river, which has created meanders, waterfalls, and syphons (in the inner part of the cave). The unsaturated zone is primarily limited to one chamber, where there are some dripping pools.

Grotta Rotolo consists of wide vertical pits and sub-horizontal passages, reaching the water table at the bottom of the final drop^44^. Within the vadose zone, several pools are present at different heights.

Grotta Puntore is located half a kilometer from the Adriatic Sea, within an area affected by seawater intrusion; this cave opens at 12 m a.s.l. as a collapse sinkhole, and it consists of a single chamber being 20 m wide and 12 m deep, almost entirely filled with breakdown deposits, hosting a brackish water lake on its northern sector.

Grotte di Castelcivita, which opens on the western slopes of the Alburni Massif, is divided into three levels: the top level represents the inactive branch; the middle one is the hydrogeologically active branch, hosting pools and lakes; the bottom level consists of permanently flooded conduits^45^. On the opposite side of the Alburni Massif is located the Grotte di Pertosa-Auletta cave: it develops along three parallel branches, where both saturated and unsaturated environments occur, including larger and smaller pools, dripping pools, and lakes. The southernmost branch is traversed by the Negro River, which flows out of the cave^46^.

Grave Grubbo cave is mainly developed along one branch within which a stream flows; there are also two other small conduits, one being totally dry and the second hosting small pools and drips only during flood periods.

Although the twelve investigated caves may not encompass all the hydrogeologic features of the macro-areas where they are located, they represent a robust compromise between sampling feasibility and representativeness of the Italian karst in terms of both hydrogeology and groundwater fauna.

### Faunal sampling

First, we carefully identified the different habitat types within each cave, including pools, streams and lakes, gours (also termed “rimstone dams”, barriers made up of calcite and other minerals being deposited around cave pools and streams). Then, we collected aquatic invertebrates using a stratified random sampling protocol, maximising sampling efforts within each habitat. For most caves, the sampling activity required advanced speleological techniques involving the use of ropes, harnesses and specific personal equipment for vertical progression in order to access the sampling sites safely. Sampling was conducted, whenever possible, in both the saturated and the unsaturated portions of the cave, and each habitat was sampled at least twice to obtain a representative sample of the corresponding invertebrate community. Faunal samples were collected and processed following a standardised protocol^47^, employing diverse sampling methods and devices based on the single habitat types.

Specifically, we sampled dripping pools by aspirating water with a 50 mL syringe or manually sieving sediments using a plankton net (60 µm mesh). In areas showing intense dripping, we placed the net directly beneath the dripping area. We sampled the freeflowing water in subterranean streams and rivulets by placing the mouth of the net against the water flow and then sieving the sediment upstream of the net mouth to dislodge the benthic and interstitial species. We used a hand net (60 µm mesh) in deeper pools and where water flow was more substantial. Sampling within the phreatic zones was conducted using the same methods and equipment just described for the vadose zones, except in the phreatic zone of the Grave Rotolo cave where we employed a 60 µm-mesh Cvetkov net^48^. The Cvetkov net was also used for sampling the “Lago della Bottiglia” lake within Grotta del Fiume.

The collected specimens were preserved in 80% ethyl alcohol and stored in the Stygobiology Laboratory at the University of L’Aquila. The Crustacea Copepoda were then sorted under a Leica M205C stereomicroscope and identified to the species/subspecies level using taxonomic keys^49–53^ and additional specialised literature. Each copepod species was further categorised as stygobite (obligate groundwater dweller) or non-stygobite (non-obligate groundwater species which are found occasionally or accidentally in groundwater).

### Data analysis

All the statistical analyses were performed through the R software version 4.2.0^54^.

#### Estimating ‘hidden’ alpha diversity and between-cave beta diversity

To assess whether the set of species recorded in the sampled caves is likely to comprehensively represent the alpha diversity of Crustacea Copepoda occurring within the Italian karst groundwaters, we used three non-parametric estimators of species richness, namely ‘Chao 2’^55^, ‘first-order Jackknife’^56^, and ‘Bootstrap’^57^, implemented within the ‘specpool’ function of the “vegan” package^58^ version 2.6–2. Further, to visualise the relationship between the number of observed species and increasing sampling effort (here intended as the number of sampled caves), we generated a Species Accumulation Curve^59^ for each of the three richness estimators, taking advantage of the 'specaccum' function from the “vegan” package. The accumulation curves were obtained through 1000 random permutations of the initial set of sites (i.e., caves) in pools of sites of increasing size (ranging from *n* = 1 to *n* = 12).

We estimated the between-cave dissimilarity of copepod assemblages by computing pairwise taxonomic beta diversity following the framework developed by Baselga^37,60^. Specifically, total pairwise beta diversity was partitioned into turnover and nestedness- resultant dissimilarity through the ‘beta.pair’ function implemented in the “betapart”^61^ R package version 1.5.6. Turnover quantifies the extent to which the difference in species composition between two sites is due to the presence of distinct species at each site. Differently, nestedness-resultant dissimilarity (hereafter, nestedness) rises when two sites share some species but one of the sites is richer in species than the other. The Sørensen dissimilarity index (β_sor_) was used as a measure of total beta diversity, turnover was measured through the Simpson dissimilarity index (β_sim_), while nestedness was measured through the corresponding index (β_sne_) described in Baselga^37^.

#### Distance-decay models

To investigate the dependence of between-cave taxonomic dissimilarity upon geographic distance, we fitted distance-decay models for total beta diversity, and for its two components separately, through the ‘decay.model’ function of the betapart package. This function permits to contrast the observed biotic dissimilarity (response variable) between two sites to the corresponding geographic distance (predictor) via a Generalized Linear Model (GLM) with normal error distribution and logarithmic link function. The Euclidean geographic distances between each pair of caves were computed through the ‘st_distance’ function of the “sf” package^62^ version 1.0–14, after projecting the original caves’ geographic coordinates (EPSG: 4326) to the ETRS89-LAEA projected reference system (EPSG: 3035). According to Gómez-Rodríguez & Baselga^39^, we tested two kinds of decay models: (i) a negative exponential model, where the predictor values (i.e., between-caves geographic distance) were left unchanged; (ii) a power-law model, fitted after log-transforming the geographic distances through natural logarithm. For each model kind, the corresponding explanatory performance was evaluated based on the model pseudo-R^2^ (${\text{pseudo}} - {\text{R}}^{{2}} = 1 - \frac{explained\,deviance}{{null\,deviance}}$) and on the *p *value resulting from the randomization algorithm implemented in the ‘decay.model’ function^39,61^, run across 1000 iterations.

#### Generalized dissimilarity models

Distance-decay models based on (generalized) linear regression have been also applied to assess whether the biotic dissimilarity between sites or regions is linked to the corresponding environmental dissimilarity^37,63^. In such applications, environmental distance between the contrasted sites/regions is usually measured as their distance along summary axes resulting from ordination techniques (e.g., Principal Component Analysis, Non-Metric Multidimensional Scaling) applied to environmental data. However, in recent years, a novel, more flexible, approach known as Generalized Dissimilarity Modelling^64^ has gained momentum and is being more and more applied to investigate the drivers behind biotic dissimilarity at various spatial scales^65^. We took advantage of the “gdm” package^66^ version 1.5.0–9.1 to fit Generalized Dissimilarity Models (GDMs) contrasting the observed between-cave beta diversity to both geographic and climatic distances.

Within GDMs, the biotic dissimilarity between sites is related to the inter-site spatial and environmental distance through a GLM with negative exponential link function (Eq. 1):

1 $$d_{ij} = 1 - e^{\eta }$$

    Where *d*_*ij*_ is the pairwise biotic dissimilarity and η is a function of inter-site environmental distance termed “predicted ecological distance”^65^. Differently from the “traditional” distance-decay models, in GDMs the predicted ecological distance is modelled based on transformed values of the predictors, resulting from a linear combination of I-spline basis functions which allow higher flexibility in modelling non-linear relationships between biotic and spatial/environmental dissimilarity^65^. Without diving into technical details, for which we refer the reader to Mokany et al.^65^, it is worth mentioning that the degree of flexibility in the parametrization of GDMs is mainly linked to: (i) the number of I-spline functions applied for transforming each predictor; (ii) the number and location of the knots along the predictors’ axes, where the knots define the portion of each predictor axis to which the single I-spline functions are applied. Here, we used the default parameters of the ‘gdm’ function from the homonymous package; specifically, three I-spline functions were used to transform each predictor, with the knots located at the minimum, median and maximum quantiles.

To quantify the climatic dissimilarity among the areas surrounding the 12 sampled caves, we took advantage of the Worldclim 2.1 online database^67^ to download gridded climate surfaces (30 arc-seconds pixel resolution, ~ 1 km^2^) representing the average values of the nineteen so-called “bioclimatic variables” (i.e., Bio1-Bio19) for the 1970–2000 period. These variables capture annual and seasonal trends related to temperature and precipitation. We chose as candidate predictors eight bioclimatic variables presumed to directly impact on subterranean environments^68^: Bio1 (Annual Mean Temperature), Bio3 (Isothermality), Bio10 (Mean Temperature of Warmest Quarter), Bio11 (Mean Temperature of Coldest Quarter), Bio12 (Annual Precipitation), Bio15 (Precipitation Seasonality), Bio18 (Precipitation of Warmest Quarter), and Bio19 (Precipitation of Coldest Quarter).

To assess whether climatic dissimilarity influences the between-cave taxonomic beta diversity in a scale-dependent manner, we computed average values of the candidate climatic predictors within four circular buffers of increasing radius around the entrance of each cave: (i) 0.5 km; (ii) 2.5 km; (iii) 5 km; (iv) 10 km. For each buffer, we performed a Variance Inflation Factor (VIF) analysis on the corresponding climatic averages through the “usdm” R package^69^ version 1.1–18, discarding the variables which attained a VIF score > 5 to avoid multicollinearity-related issues in model calibration^70^. Successively, we performed 1000 GDM fitting iterations: for each iteration, we randomly selected 85% of the 66 cave pairs (*n* = 56) to populate the training data table; these data were then used to calibrate a separate GDM for each combination of dissimilarity metric (i.e., β_sor_, β_sim_, β_sne_) × buffer radius. Within each GDM, the dissimilarity metric represented the response variable, while between-cave geographic distance and between-cave differences in the average value of each retained climatic variable within the considered buffer represented the explanatory variables. We then assessed the explanatory performance of each model based on the corresponding explained deviance (on training data), and the model predictive performance by computing the Root Mean Squared Error (RMSE) of its predictions on the withheld cave pairs (*n* = 10).

For each fitted GDM, we also partitioned, through the ‘gdm.partition.deviance’ function, the portion of explained deviance attributable to three sets of variables (and pairwise combinations thereof): (i) “Dist”, including only pairwise geographic distance; (ii) “Prec”, comprising the precipitation-related variables Bio12, Bio15, Bio18 and Bio19; (iii) “Temp”, including the temperature-related variables Bio3, Bio10 and Bio11 (Bio1 was discarded, after the VIF, for all the four buffer radii).

We then investigated possible statistically significant differences (in terms of explained deviance, RMSE and deviance partitioning) among the GDMs fitted on climatic averages from the four distinct buffer radii by performing Kruskal–Wallis Rank Sum Tests, followed by post-hoc one-tailed Pairwise Wilcoxon Rank Sum Tests with Benjamini & Hochberg^71^ *p *value correction. We used non-parametric tests because preliminary data exploration^72^ highlighted, for several dissimilarity metric × buffer radius combinations, the lack of fundamental assumptions of parametric tests (e.g., normality of residuals, homoscedasticity). For all these tests, the threshold for Type I error (i.e., “alpha-level”) was set to *p *value  < 0.05.

Finally, we fitted a full-data GDM for each dissimilarity metric × buffer radius combination. From these full-data models, we inspected: (i) the distribution of predicted *versus* observed dissimilarity values; (ii) the relative importance of each explanatory variable, assessed through the permutation algorithm implemented in the ‘gdm.VarImp’ function; (iii) the I-spline curves corresponding to the top-three variables in terms of relative importance.

**Figure 2:**
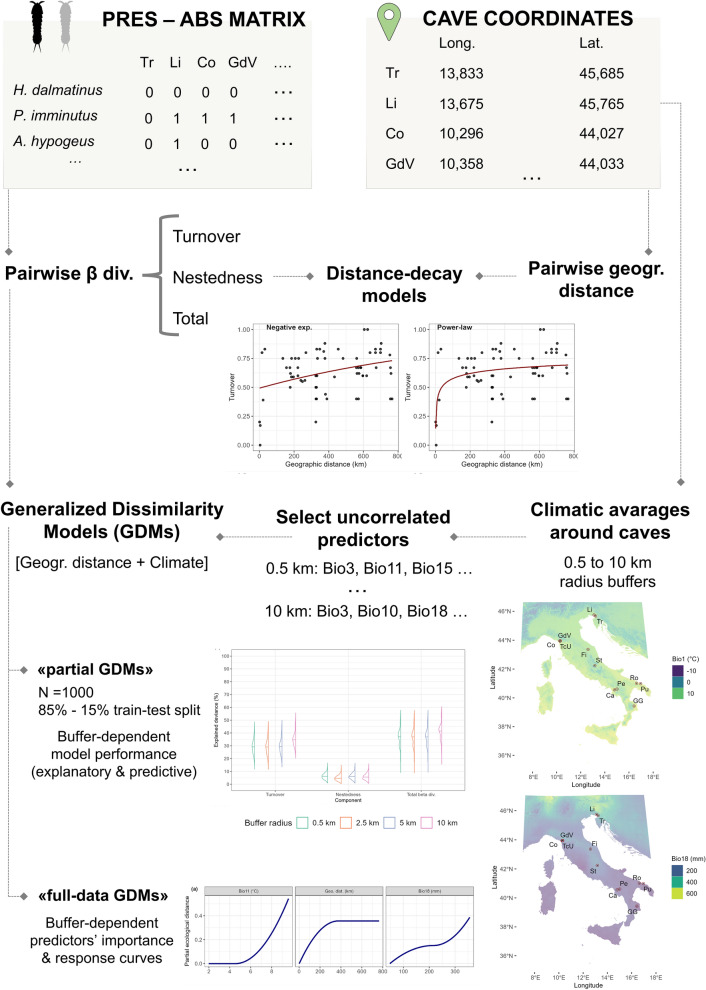
Flowchart summarising the different analytical steps taken to investigate the contribution of geographical distance and surface climatic conditions to between-cave taxonomic beta diversity.

A flowchart summarising the workflow designed to conduct the set of analyses described above is shown in Fig. 2.

## Results

Out of the 71 copepod species recorded in the 12 sampled caves, 46 were stygobites and 25 non-stygobites (Supplementary Materials Table S1). Among the non-stygobites, *Paracyclops imminutus* was by far the most widespread as it was recorded in 11 caves, followed by *Bryocamptus echinatus* which was found in 8 caves. Worth mentioning, 45 out of the 71 species were collected in a single cave each. Grotte di Pertosa-Auletta (Pe) showed the highest alpha diversity (22 species), followed by Grotte di Stiffe (St) with 18 species. Grotta Lindner (Li) and Tana che Urla (TcU) showed the lowest number of recorded species (5), followed by Grave Rotolo (Ro) and Grotta del Vento (GdV) hosting 6 species each. Some of the species being unique to single caves still lack scientific description, such as a new species of *Hesperocyclops* from Grave Rotolo and a new species of *Pseudectinosoma* from Grotta Puntore (Pu).

Despite the relatively high number of recorded species, all three non-parametric richness estimators suggested that more species may have been found if additional caves were sampled. Indeed, the ratio between observed and estimated richness was as low as 40.8% according to the Chao2 estimator which, however, also showed a high standard error (Supplementary Material Table S2); higher ratios between observed and estimated richness emerged for the first-order Jackknife (63.3%) and the Bootstrap (88.1%) estimators (Supplementary Material Table S2). The accumulation curves obtained using the three estimators were almost identical and none of them approached an asymptotic trend (Supplementary Material Fig. S1).

Total between-cave beta diversity was relatively high for most pairs of caves (Supplementary Material Table S3), with β_sor_ values lower than 0.3 emerging only between the three caves located in the Apuan Alps [i.e., Antro del Corchia (Co), Grotta del Vento (GdV), and Tana che Urla (TcU)]. Further, overall beta diversity (mean β_sor_ ± SD = 0.64 ± 0.31; median β_sor_ = 0.76) was mostly driven by turnover (mean β_sim_ ± SD = 0.53 ± 0.29; median β_sim_ = 0.60), with far lower nestedness-resultant dissimilarity (mean β_sne_ ± SD = 0.11 ± 0.11; median β_sne_ = 0.07). A preliminary visual comparison between dissimilarity values and geographic distances (Supplementary Material Tables S3, S4) revealed that relatively close caves may be noticeably dissimilar in terms of copepod assemblages, while caves located hundreds of kilometres apart may have quite a low species turnover. For instance, Abisso di Trebiciano (Tr) and Grotta Lindner (Li), which are just 15 km distant, showed a high beta diversity (β_sor_ = 0.86) and, most notably, this was almost totally driven by turnover (β_sim_ = 0.8). Differently, Grotte di Stiffe (St) and TcU, which are located 325 km apart, showed one of the lowest turnover values (β_sim_ = 0.2), with beta diversity in this case being primarily driven by nestedness of the TcU assemblage within that of St (β_sne_ = 0.48).

**Figure 3:**
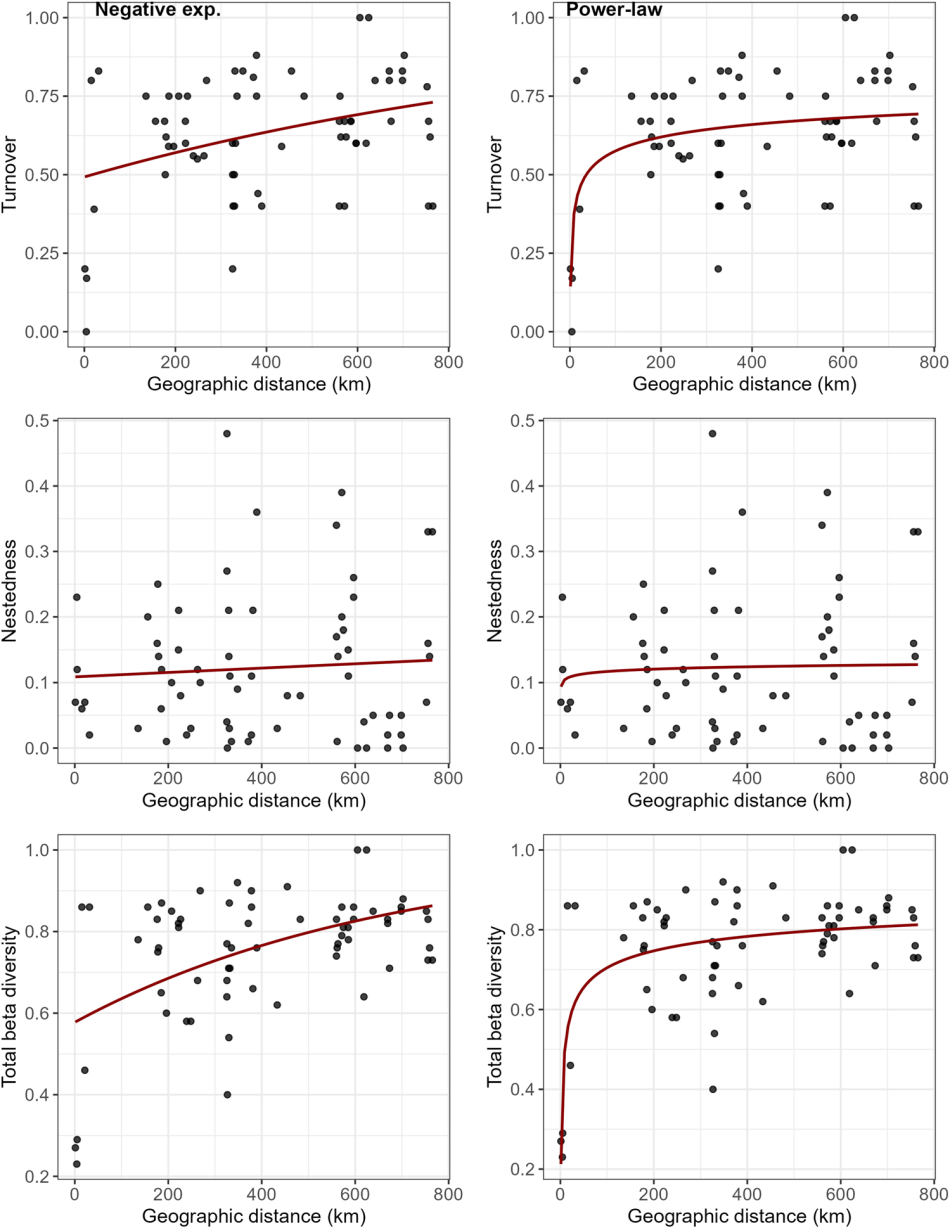
Distance-decay curves resulting from the distance-decay models based on either a negative exponential function (left plots) or a power-law function (right plots), fitted for between-cave turnover, nestedness and total beta diversity. Black dots showed the observed beta diversity—geographic distance pairs of values.

Both the negative exponential and the power-law distance-decay models showed statistically significant explanatory performance for both turnover and total beta diversity, but not for nestedness (Supplementary Material Table S5). The distance-decay curves resulting from the power-law model showed a better fit to observed values compared to the ones resulting from the negative exponential model, though both of them either underestimated or overestimated dissimilarity for some pairs of caves (Fig. 3).

**Figure 4:**
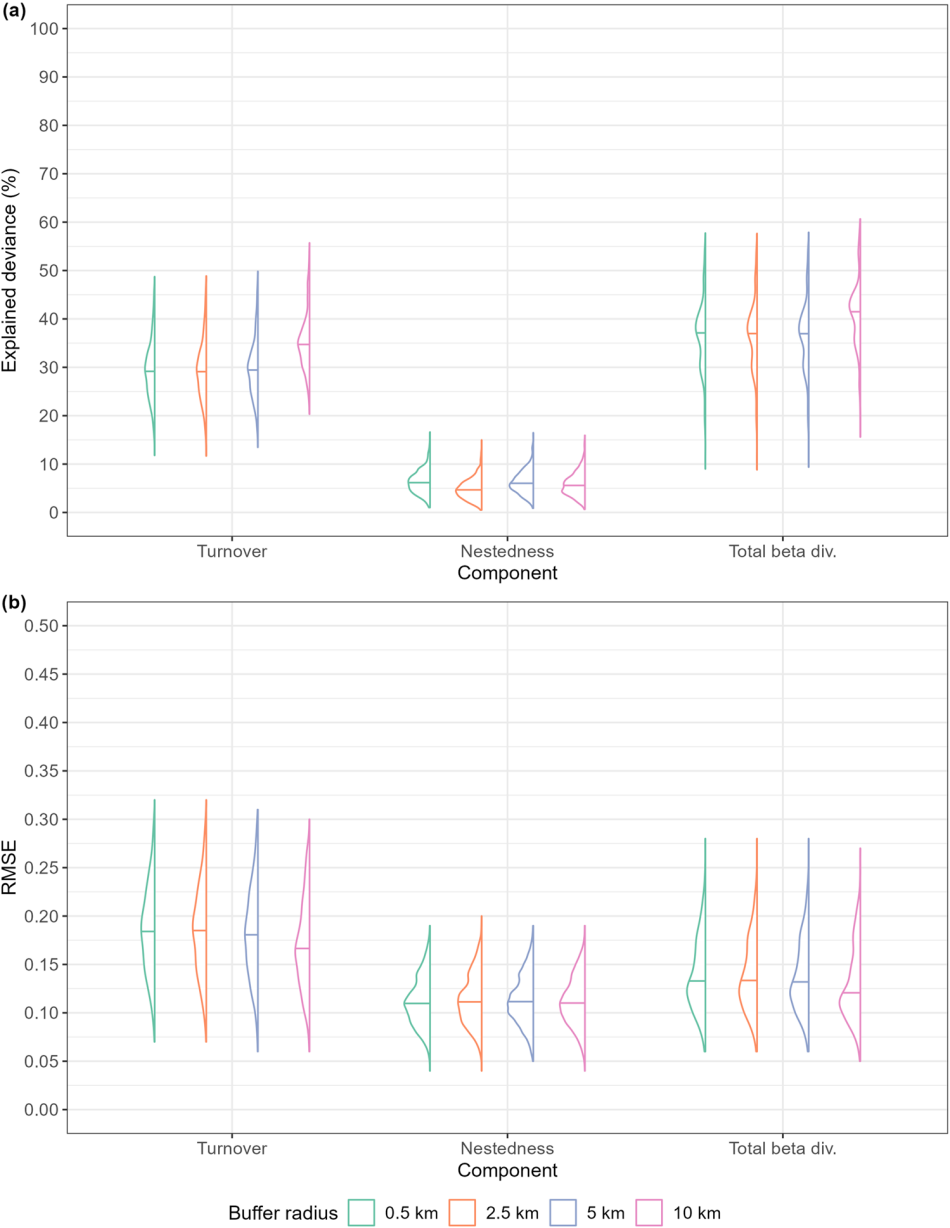
Half-violin plots showing variation in (**a**) explained deviance, and (**b**) RMSE on test data, extracted from the Generalized Dissimilarity Models fitted using climatic averages computed over four distinct buffer radii around cave locations (0.5 km, 2.5 km, 5 km and 10 km) as explanatory variables (along with between-cave geographic distance). The horizontal bar within each half-violin indicates the median.

Whatever the extent of the buffer over which the climatic averages were computed, the explanatory performance of the GDMs fitted with nestedness as response variable was far lower (median explained deviance < 10%) than that of the models fitted for turnover and total beta diversity (Fig. 4), thus mirroring the results of the distance-decay models.

The GDMs fitted on climatic averages computed over circular buffers of increasing radius around the caves significantly differed in terms of both explained deviance (Turnover: χ^2^ = 627.70, DF = 3, *p *value  < 0.001; Nestedness: χ^2^ = 249.38, DF = 3, *p *value  < 0.001; Total beta diversity = 305.74, DF = 3, *p *value  < 0.001) and RMSE on test data (Turnover: χ^2^ = 71.65, DF = 3, *p *value  < 0.001; Total beta diversity = 57.58, DF = 3, *p *value  < 0.001), except for RMSE of models built for nestedness (χ^2^ = 4.26, DF = 3, *p *value  = 0.24). For turnover and total beta diversity, the GDMs fitted using the 10 km-radius buffer showed higher explanatory (i.e., higher explained deviance) and predictive (i.e., lower RMSE) performance compared to the models fitted using the lower size buffers (*p* < 0.001 for all the pairwise comparisons in the post-hoc tests). For nestedness, instead, no consistent scale-dependent differences emerged in terms of either explanatory or predictive performance (Supplementary Materials Table S6).

**Figure 5:**
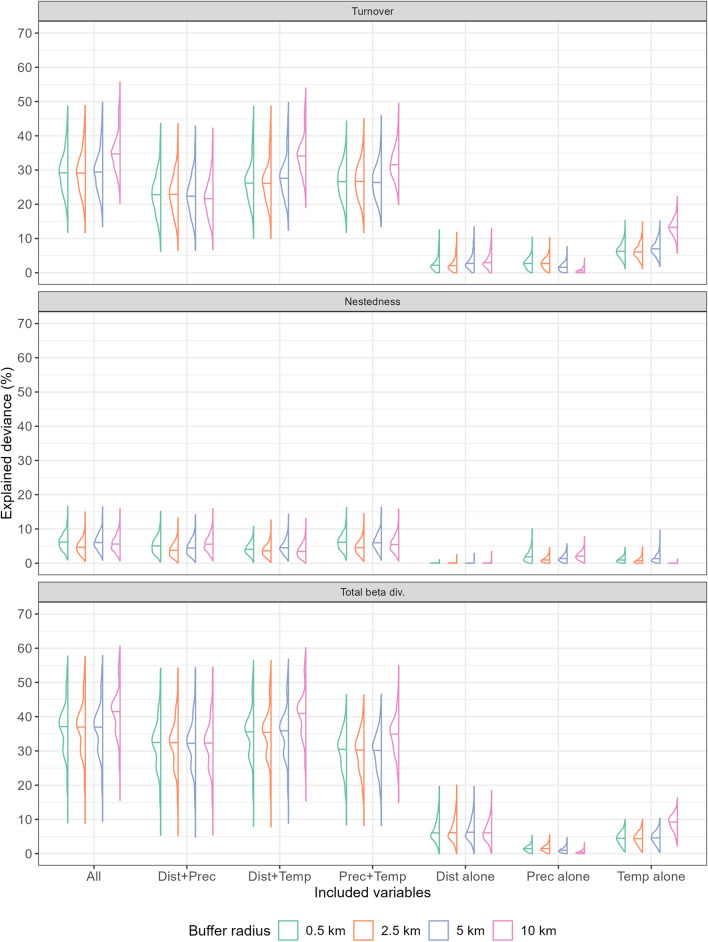
Half-violin plots summarising the results of the deviance partitioning algorithm applied to the Generalized Dissimilarity Models fitted using climatic averages computed over four distinct buffer radii around cave locations (0.5 km, 2.5 km, 5 km and 10 km) as explanatory variables (along with between-cave geographic distance). The horizontal bar within each half-violin indicates the median. “Dist” = geographic distance; “Temp” = temperature-related variables; “Prec” = precipitation-related variables.

Focusing on turnover and total beta diversity, buffer size also influenced the percentage of deviance explained by the different sets of variables (*p* < 0.001 in the Kruskal–Wallis Rank Sum Tests), except within the GDMs targeting total beta diversity and fitted upon “Dist” only (χ^2^ = 5.141, DF = 3, *p *value  = 0.16) and “Dist + Prec” (χ^2^ = 0.982, DF = 3, *p *value  = 0.806). Post-hoc tests confirmed significantly higher explained deviance using a 10 km-radius compared to the other three radii for the GDMs including temperature variables, namely those fitted only on “Temp”, “Dist + Temp” and “Prec + Temp” (Supplementary Materials Table S7). Further, the GDMs targeting turnover and fitted only on “Temp” variables had higher explanatory performance than those fitted using only geographic distance or “Prec” variables, and their median explained deviance increased about twicefold when using a 10 km-radius buffer (Fig. 5). For total beta diversity, GDMs fitted only on “Dist” explained as much deviance as those fitted on “Temp”, except when temperature averages were computed over a 10 km-radius buffer (Fig. 5). However, none of the models fitted on a single set of variables showed explained deviance higher than 15%. The GDMs fitted on coupled sets of variables generally explained less deviance than models including all the variables, but such difference was slight for “Dist + Temp” models fitted using the 10 km-radius buffer (Fig. 5).

GDMs fitted on all the 66 cave pairs (i.e., “full-data models”) confirmed that computing climatic averages over a wider area around cave locations increased model fit on observations, for both turnover and total beta diversity. Indeed, Spearman’s correlation between predicted and observed dissimilarity values increased from *r* = 0.4 with 0.5 km-radius buffer to *r* = 0.5 with 10 km-radius buffer for turnover, and from *r* = 0.32 with 0.5 km-radius buffer to *r* = 0.45 with 10 km-radius buffer for total beta diversity (Supplementary Materials Figs. S2, S4). Contrarily, the low correlation between predicted and observed values of nestedness further decreased when using the 10 km-radius buffer (Supplementary Materials Fig. S3).

“Temp” variables (i.e., Bio10, Bio11) consistently emerged as the top-contributing ones, followed by geographic distance, within the full-data GDMs fitted with turnover as response variable (Table 2). Differently, geographic distance was the most influential predictor for total beta diversity, except within the full-data GDM fitted using the 10 km-radius buffer. In line with the results of the deviance partitioning analysis conducted on the 1000 partial GDMs (i.e., those fitted on 85% random samples of the cave pairs and validated on the remaining 15%), the relative importance of seasonal temperature averages noticeably increased within the full-data GDMs fitted using the 10 km-radius buffer, for both turnover and total beta diversity (Table 2). In the latter models, Bio11 was excluded from model fitting due to multicollinearity emerging in the VIF, then being replaced by Bio10 as the leading variable.

**Table 2:** Top-three variables, in terms of relative contribution, within the full-data GDMs fitted for turnover and total beta diversity using the four distinct buffer radii for spatial averaging of climate-related variables.

Turnover			Total beta diversity		
Buffer radius	Variable	Relative contribution (%)	Buffer radius	Variable	Relative contribution (%)
0.5 km	Bio11	19.4	0.5 km	Geo. dist	17.4
	Geo. dist	7.7		Bio11	10.8
	Bio18	6.9		Bio19	1.9
2.5 km	Bio11	19.2	2.5 km	Geo. dist	17.4
	Geo. dist	8.1		Bio11	10.5
	Bio18	6.7		Bio19	2.3
5 km	Bio11	19.9	5 km	Geo. dist	17.8
	Geo. dist	9.7		Bio11	10.7
	Bio18	3.6		Bio18	1.7
10 km	Bio10	37.9	10 km	Bio10	20.1
	Geo. dist	10.2		Geo. dist	14.9
	Bio18	2.5		Bio18	1.5

**Figure 6:**
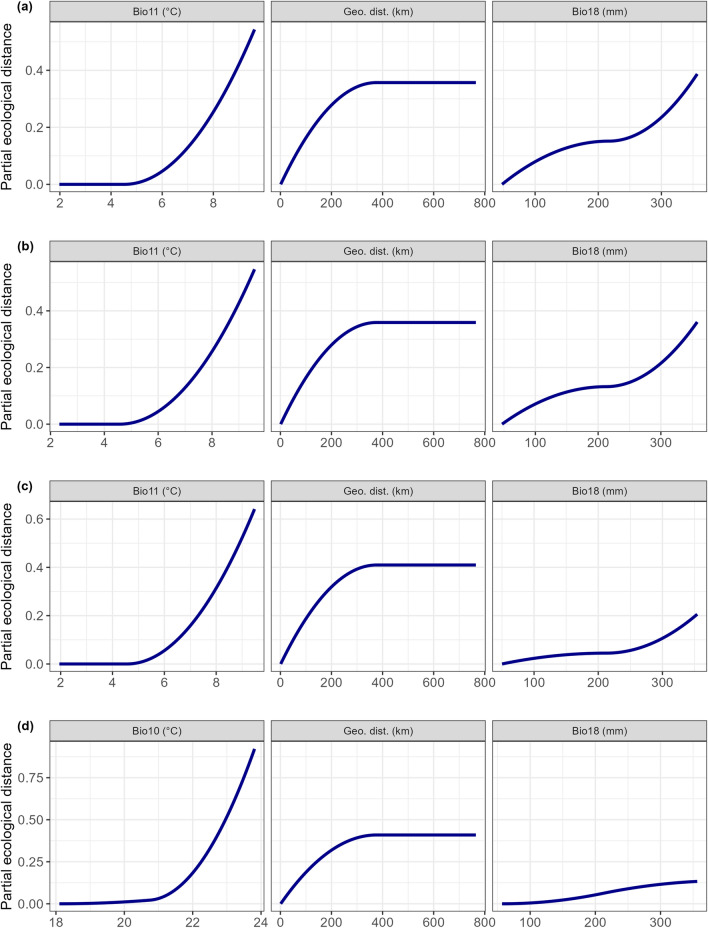
I-spline curves extracted from the full-data GDMs fitted with turnover as response variable, and including as explanatory variables climatic averages computed over a buffer around the caves’ entrance whose radius measured: (**a**) 0.5 km, (**b**) 2.5 km, (**c**) 5 km, and (**d**) 10 km. The curves show the relationship between modelled partial ecological distance and the values of the top-three variables in terms of relative contribution to the considered GDM. In each row, the order of the plots reflects the relative contribution of the variables, with the top-contributing variable on the left.

The I-spline curves extracted from the full-data GDMs confirmed the strong contribution of temperature gradients to the predicted dissimilarity in terms of turnover (Fig. 6): indeed, an exponential growth in the modelled partial ecological distance emerged with increasing temperature for both Bio11 and Bio10, while the curves obtained for the other predictors showed logarithmic (for geographic distance) or sigmoidal (for “Prec” variables, i.e. Bio18 and Bio19) trends, with far lower maximum values. For total beta diversity, the I-spline drawn for geographic distance still showed a logarithmic trend but with a far steeper increase in partial ecological distance up to 300 km and a highest peak (Supplementary Materials Fig. S5). Nonetheless, changes along the gradient of Bio10 translated into increases in partial ecological distance far more rapidly than for geographic distance within the full-data model fitted with the 10 km-radius buffer (Supplementary Materials Fig. S5).

## Discussion

The twelve caves showed a notable variety of copepod species. Species fully dependent on groundwater (i.e., stygobites) and species occasionally or accidentally entering cave waters from the surface (i.e., non-stygobites) were both well represented, though with the primacy of the obligate groundwater dwellers. Overall, we identified 71 copepod species in the collected samples and some of the sampled caves hosted more than 15 species. Nonetheless, the implemented richness estimators indicated that the set of recorded species was likely not exhaustive in terms of copepod alpha diversity across the investigated karst macro-areas. This is in line with the modern view of subterranean environments being far richer in life than previously thought^9,18,73^, and it further reinforces the recent claims of the scientific community about the urgency of filling the existing knowledge gaps about subterranean biodiversity^6,74^. Moreover, the high incidence of spot endemics (23 stygobitic species) and of rare—in terms of frequency of occurrence—species (5 stygobitic species) is likely the main factor that determined a long upward slope to the asymptote in the obtained species accumulation curves^75^.

Along with a high alpha diversity, we were able to highlight a striking between-cave taxonomic dissimilarity (i.e., beta diversity) which was driven mainly by pure species replacement, in line with what has been derived for the European groundwater fauna by Zagmajster et al.^76^, from the regional to the continental scale. The dominance of turnover over nestedness-resultant dissimilarity (i.e., dissimilarity due to ordered species loss) in shaping beta diversity is emerging in numerous other ecosystems across a wide variety of spatial scales. In a recent meta-analysis including 99 studies conducted in various regions across the globe, Soininen et al.^77^ showed that available evidence points to a major role of turnover particularly at lower latitudes and with increasing extent of the studied area, while nestedness-resultant dissimilarity generally increases towards the poles. This trend is likely due to stronger environmental filtering at higher latitudes, driven by factors such as paleoclimatic events (e.g., Quaternary glaciations) and harsher current conditions (e.g., longer freezing periods and lower primary productivity).

Across the European continent, similar patterns were retrieved for a wide array of taxa, including beetles^37,78^, mammals^79^, parasitic trematodes^80^, aquatic plants and cladocerans^81^, freshwater fishes^82^, and groundwater crustaceans^33^. In such studies, the relative contribution of spatial (i.e., geographic distance) versus environmental (e.g., climate, land cover, soil composition) factors on the ratio between turnover and nestedness also varied depending on the target taxa. For instance, Heino et al.^78^ found that overall beta diversity among northern European regions was similar between ground beetles and diving beetles; however, compositional dissimilarity linked to richness differences (corresponding to nestedness-resultant dissimilarity) was higher than turnover for the former group, and vice versa for the latter. For ground beetles, the authors linked the preponderance of nestedness, and the major contribution of geographic distance to the observed dissimilarity, to the Quaternary glaciations which have determined regional extinctions in northern areas and to biogeographical barriers hampering subsequent re-colonizations. Differently, the higher turnover emerging for diving beetles was more strongly related to temperature gradients than to spatial isolation, suggesting that environmental filtering rather than dispersal barriers shaped current beta diversity patterns for this group. In southern Europe, however, the dominance of pure species replacement has emerged for various taxa with widely diverging life-history traits, dispersal capability and evolutionary history^33,79,81^. This pattern has been generally attributed to the higher historical climatic stability of southern Europe compared to boreal regions, which would have favoured speciation events over long periods devoid of dramatic climatic changes causing mass extinctions.

Focusing on groundwater crustaceans, copepods included, Zagmajster et al.^33,76^ confirmed higher turnover values at lower latitudes, a major contribution of turnover over nestedness to total beta diversity, and significant linear correlations (positive for turnover, negative for nestedness) with geographic distance. At a far finer spatial scale, a study conducted on copepod assemblages populating a set of thirty groundwater-fed springs from Central Italy^36^ highlighted a high nestedness-resultant dissimilarity, which was also positively correlated to between-spring geographic distance; such a correlation with geographic distance did not emerge, instead, for the turnover component. However, in both these studies, the authors did not implement any analytical tool permitting to explicitly discriminate the proportion of dissimilarity being purely attributable to geographic distance from that driven by environmental gradients.

The distance-decay models we implemented, using either a negative exponential function or a power-law function, showed that total beta diversity and turnover were significantly related to between-cave geographic distance, while nestedness was not. However, distance-decay curves did not perfectly fit the observed dissimilarity values, due to the fact that the relationship between geographic distance and faunal dissimilarity was not always positive. Indeed, some caves far apart from each other shared more species of copepods than neighbouring caves. For instance, the Tana che Urla and the Grotte di Stiffe caves, which are 325 km apart, share 3 non-stygobitic species (*Bryocamptus echinatus*, *Bryocamptus zschokkei* and *Paracyclops imminutus*) and the wide-ranging stygobitic harpacticoid *Elaphoidella phreatica*, leading to a low turnover (β_sim_ = 0.2). Contrarily, the Abisso di Trebiciano and the Grotta Lindner caves, belonging to the same hydrogeological unit (Classical Karst^83^) and being closer than 20 km, shared the non-stygobitic species *Bryocamptus echinatus* only. In this case, a high between-cave turnover (β_sim_ = 0.8) emerged because Abisso di Trebiciano hosted 3 stygobitic (*Troglodiaptomus sketi*, *Diacyclops charon* and *Elaphoidella jeanneli*) and 4 non-stygobitic (*Acanthocyclops robustus*, *Macrocyclops albidus*, *Megacyclops viridis* and *Bryocamptus zschokkei*) species which were not found in Grotta Lindner, while the latter hosted 3 stygobites (*Acanthocyclops hypogeus*, *Elaphoidella elaphoides* and *Elaphoidella phreatica*) and the non-stygobite *Paracyclops imminutus* which were absent from Abisso di Trebiciano. These examples suggest that the actual degree of between-cave species replacement is determined by both the stygobitic and the non-stygobitic species. Specifically, low species turnover between distant caves could be primarily related to non-stygobitic, often widely distributed, species that have established populations (most likely temporarily) within the caves under consideration. On the other hand, high turnover between close caves was largely driven by the stygobitic copepods, which are often unable to cross the rocky boundaries between even adjacent caves due to their low potential for dispersal, and thus usually respond to the rule “each species per each cave”^13,18,74^. Along with dispersal barriers related to the lithology of the areas where the caves open^14^, changes in groundwater faunistic assemblages over regional spatial scales could also derive from differences in the size, spatial arrangement and heterogeneity of the different habitat types and/or in the physical–chemical conditions (e.g., pH, electric conductivity) characterising distinct caves belonging to adjacent—or even to the same—hydrogeological units^84^.

By fitting Generalized Dissimilarity Models (GDM) fed with information about surface climatic conditions around the sampled caves, we found that a major portion of the observed between-cave compositional dissimilarity is explained by a mix of spatial (i.e., geographic distance) and climatic factors. Indeed, quarterly temperature averages (i.e., Bio10 and Bio11) consistently emerged as the most influential explanatory variables in the GDMs fitted for between-cave turnover. With respect to total beta diversity, Bio10 was still by far the most influential variable when sampling climate data over a 10 km-buffer radius, but geographic distance showed a not negligible influence in the corresponding model. Therefore, environmental filtering, primarily related to thermal ranges, appeared to mainly determine the set of species being unique to the different caves, and thus between-cave turnover. This may derive from the combination of two processes: (i) differences in temperature averages between regions may translate into differences in the timing and amount of groundwater recharge following ice and snow melting, along with rainfall, which in turn affects the degree to which non-stygobitic copepods can move in the groundwater through the hydrologic continuum between surface water and groundwater^85^; (ii) since temperature within caves is generally correlated to surface mean annual temperature^86^, temperature patterns in the neighbourhoods of the different caves may directly translate into thermal differences in cave waters, thus shaping the corresponding assemblages based on the thermal niche breadth of the species forming the respective regional pools^87,88^.

Further, we found that the extent over which climate averages were computed significantly influenced, for both turnover and total beta diversity, the goodness-of-fit of the GDMs, their predictive performance and the relative importance of temperature-related explanatory variables. Indeed, when increasing the radius of the buffer around the sampled caves up to 10 km, the proportion of explained deviance and the correlation between observed and predicted dissimilarity values increased, the RMSE of model predictions on the test data decreased, and Bio10 became by far the most influential variable. Additionally, the variance partitioning experiment clearly showed that temperature-related variables provided the greatest amount of information, when used in isolation or coupled with geographic distance, to model between-cave partial ecological distance. Again, the contribution of temperature-related variables within the GDMs fitted on subsets of the available explanatory variables significantly increased when using a 10 km-radius buffer. These patterns may be linked to the fact that the aquatic habitats of caves are replenished by surface rainfall and temperature-driven ice and snow melting over recharge areas which usually span dozens-to-hundreds of kilometres and that are often located far from the caves’ openings^14,89^. Thus, extending the area from which data are acquired should provide more information about how differences in regional climate affect the aquifers' recharge in the different caves, and this in turn should increase our ability to isolate climatic correlates of between-cave biotic dissimilarity. Our findings also recall those from Keil et al.^90^ who showed that, over regional extents (i.e., single countries or more restricted areas), climatic factors were more important than land cover and geographic distance in explaining pairwise species turnover for various taxonomic groups in Europe, and that the relative contribution of climate increased at coarser grid resolutions. The residual unexplained deviance in our models, namely the portion of between-cave beta diversity which could not be directly linked either to climatic averages or to geographic distance, suggests that the inclusion of some of the local-scale factors cited above (e.g., habitat size and heterogeneity, physical–chemical parameters) would be recommendable to fully understand the drivers behind the composition of crustacean assemblages in cave waters. However, while measuring differences in average physical–chemical parameters between distinct caves is rather straightforward, quantifying between-cave differences in habitat heterogeneity is a far more complex operation, also affected by subjectivity in classifying the single habitat patches within the compared caves.

In conclusion, this study highlighted that Italian karst caves harbour a rich and diverse array of copepod species, including several stygobitic and narrow-ranging species. Further, we demonstrated that copepod assemblages populating a set of caves along a latitudinal gradient spanning the Italian peninsula were noticeably dissimilar from one another, with turnover rather than nestedness driving most of the observed beta diversity. This is also important from a conservation perspective because the high species turnover implies that environmental degradation within a single cave may lead to the loss of unique species not occurring elsewhere, similarly to what has emerged from a recent study performed on groundwater-fed karst springs^41^. Additionally, we showed that geographical distance had a not negligible yet secondary role in shaping the between-cave dissimilarity of copepod assemblages in our studied caves, with surface temperature patterns having the greatest influence, particularly on species replacement. Finally, we showed that widening the spatial extent from which climatic data were retrieved in the surroundings of the target caves increased the ability of Generalized Dissimilarity Models to explain the observed beta diversity patterns, along with the relative importance of temperature-related variables. A limitation of the present study about this latter finding is that, by using circular buffers to compute climatic averages, we did not take into account the inherent anisotropy of the environmental gradients affecting the groundwater recharge dynamics (e.g., altitude and geomorphology influencing the direction from which water flows towards a given cave). Further, the possible presence of cryptic (i.e., with very similar phenotypes yet genetically distinct) groundwater-dwelling species may affect the estimates of between-cave beta diversity and its components, a risk which could be lowered by coupling molecular species delimitation methods to the morphology- and ecology-based taxonomic assessment^91^. Future studies replicating our analyses by taking into account these points, also focusing on caves from other regions, broader spatial extents and/or different subterranean taxa, would increase our understanding of the drivers behind the structuring and diversification of groundwater communities.

## Data availability

Geographic coordinates of the sampled caves are provided in Table 1. Presence-absence data of the recorded copepod species are provided in Supplementary Materials Table S1. Climate data used to fit the Generalized Dissimilarity Models are freely available from the Worldclim online repository (https://www.worldclim.org/).

### Supplementary Information

Supplementary Information.

## References

[1] Marmonier P, Galassi DMP, Korbel K, Close M, Datry T, Karwautz C, Malard F, Griebler C, Rétaux S (2023). Groundwater biodiversity and constraints to biological distribution. Groundwater Ecology and Evolution.

[2] Iannella M, Fiasca B, Di Lorenzo T, Di Cicco M, Biondi M, Mammola S, Galassi DMP (2021). Getting the ‘most out of the hotspot for practical conservation of groundwater biodiversity. Glob. Ecol. Conserv..

[3] Sánchez-Fernández D, Galassi DMP, Wynne JJ, Cardoso P, Mammola S (2021). Don’t forget subterranean ecosystems in climate change agendas. Nat. Clim. Change.

[4] Ficetola GF, Canedoli C, Stoch F (2019). The Racovitzan impediment and the hidden biodiversity of unexplored environments. Conserv. Biol..

[5] Mammola S, Lunghi E, Bilandžija H, Cardoso P, Grimm V, Schmidt SI, Hesselberg T, Martínez A (2021). Collecting eco-evolutionary data in the dark: Impediments to subterranean research and how to overcome them. Ecol. Evolut..

[6] Mammola S, Meierhofer MB, Borges PA, Colado R, Culver DC, Deharveng L, Delić T, Di Lorenzo T, Dražina T, Ferreira RL, Fiasca B (2022). Towards evidence-based conservation of subterranean ecosystems. Biol. Rev..

[7] Colado R, Abellán P, Pallarés S, Mammola S, Milione R, Faille A, Fresneda J, Sánchez-Fernández D (2023). A dark side of conservation biology: Protected areas fail in representing subterranean biodiversity. Insect Conserv. Divers..

[8] Camacho A, Valdecasas A, Rodríguez J, Cuezva S, Lario J, Sánchez-Moral S (2006). Habitat constraints in epikarstic waters of an Iberian Peninsula cave system. Ann. Limnol. Int. J. Limnoogy.

[9] Pipan T, Culver DC (2007). Epikarst communities: Biodiversity hotspots and potential water tracers. Environ. Geol..

[10] Zagmajster M, Culver DC, Sket B (2008). Species richness patterns of obligate subterranean beetles (Insecta: Coleoptera) in a global biodiversity hotspot–effect of scale and sampling intensity. Divers. Distrib..

[11] Trontelj P, Borko Š, Delić T (2019). Testing the uniqueness of deep terrestrial life. Sci. Rep..

[12] Iannella M, Fiasca B, Di Lorenzo T, Biondi M, Di Cicco M, Galassi DMP (2020). Jumping into the grids: Mapping biodiversity hotspots in groundwater habitat types across Europe. Ecography.

[13] Iannella M, Fiasca B, Di Lorenzo T, Biondi M, Di Cicco M, Galassi DMP (2020). Spatial distribution of stygobitic crustacean harpacticoids at the boundaries of groundwater habitat types in Europe. Sci. Rep..

[14] Vaccarelli I, Cerasoli F, Mammola S, Fiasca B, Di Cicco M, Di Lorenzo T, Stoch F, Galassi DM (2023). Environmental factors shaping copepod distributions in cave waters of the Lessinian unsaturated karst (NE-Italy). Front. Ecol. Evol..

[15] Galassi DMP, Stoch F, Fiasca B, Di Lorenzo T, Gattone E (2009). Groundwater biodiversity patterns in the Lessinian massif of northern Italy. Freshw. Biol..

[16] Galassi DMP, Fiasca B, Di Lorenzo T, Montanari A, Porfirio S, Fattorini S (2017). Groundwater biodiversity in a chemoautotrophic cave ecosystem: How geochemistry regulates microcrustacean community structure. Aquat. Ecol..

[17] Di Lorenzo T, Cipriani D, Fiasca B, Rusi S, Galassi DMP (2018). Groundwater drift monitoring as a tool to assess the spatial distribution of groundwater species into karst aquifers. Hydrobiologia.

[18] Galassi DM, Huys R, Reid JW (2009). Diversity, ecology and evolution of groundwater copepods. Freshw. Biol..

[19] Borko Š, Trontelj P, Seehausen O (2021). A subterranean adaptive radiation of amphipods in Europe. Nat. Commun..

[20] Mammola S (2019). Finding answers in the dark: Caves as models in ecology fifty years after Poulson and White. Ecography.

[21] Parise M, Gabrovsek F, Kaufmann G, Ravbar N, Parise M, Gabrovsek F, Kaufmann G, Ravbar N (2018). Recent advances in karst research: from theory to fieldwork and applications. Advances in Karst Research: Theory, Fieldwork and Applications.

[22] Culver DC, Pipan T (2009). The Biology of Caves and Other Subterranean Habitats.

[23] Pipan T, Culver DC (2013). Forty years of epikarst: What biology have we learned?. Int. J. Speleol..

[24] Griebler C, Avramov M (2015). Groundwater ecosystem services: A review. Freshw. Sci..

[25] Ferreira RL, Bernard E, da Cruz Júnior FW, Piló LB, Calux A, Souza-Silva M, Barlow J, Pompeu PS, Cardoso P, Mammola S, García AM (2022). Brazilian cave heritage under siege. Science.

[26] North LA, van Beynen PE, Parise M (2009). Interregional comparison of karst disturbance: West-central Florida and southeast Italy. J. Environ. Manag..

[27] Bakalowicz M, White WB, Culver DC, Pipan T (2019). Epikarst. Encyclopedia of Caves.

[28] Danielopol DL, Pospisil P, Rouch R (2000). Biodiversity in groundwater: A large-scale view. Trends Ecol. Evolut..

[29] Culver DC, Pipan T, Schneider K (2009). Vicariance, dispersal and scale in the aquatic subterranean fauna of karst regions. Freshw. Biol..

[30] Fattorini S, Fiasca B, Di Lorenzo T, Di Cicco M, Galassi DMP (2020). A new protocol for assessing the conservation priority of groundwater-dependent ecosystems. Aquat. Conserv. Mar. Freshw. Ecosyst..

[31] Dole-Olivier M-J, Malard F, Martin D, Lefébure T, Gibert J (2009). Relationships between environmental variables and groundwater biodiversity at the regional scale. Freshw. Biol..

[32] Hahn HJ, Fuchs A (2009). Distribution patterns of groundwater communities across aquifer types in south-western Germany. Freshw. Biol..

[33] Zagmajster M, Eme D, Fišer C, Galassi D, Marmonier P, Stoch F, Cornu JF, Malard F (2014). Geographic variation in range size and beta diversity of groundwater crustaceans: Insights from habitats with low thermal seasonality. Glob. Ecol. Biogeogr..

[34] Fattorini S, Lombardo P, Fiasca B, Di Cioccio A, Di Lorenzo T, Galassi DMP (2017). Earthquake-related changes in species spatial niche overlaps in spring communities. Sci. Rep..

[35] Devitt AM, Wright DC, Cannatella DM, Hillis DM (2019). Species delimitation in endangered groundwater salamanders: Implications for aquifer management and biodiversity conservation. Proc. Natl. Acad. Sci. U. S. A..

[36] Fattorini S, Borges PAV, Fiasca B, Galassi DMP (2016). Trapped in the web of water: Groundwater-fed springs are island-like ecosystems for the meiofauna. Ecol. Evolut..

[37] Baselga A (2010). Partitioning the turnover and nestedness components of beta diversity. Glob. Ecol. Biogeogr..

[38] Fitzpatrick MC, Sanders NJ, Normand S, Svenning JC, Ferrier S, Gove AD, Dunn RR (2013). Environmental and historical imprints on beta diversity: Insights from variation in rates of species turnover along gradients. Proc. R. Soc. B Biol. Sci..

[39] Gómez-Rodríguez C, Baselga A (2018). Variation among European beetle taxa in patterns of distance decay of similarity suggests a major role of dispersal processes. Ecography.

[40] Ambrožová L, Čížek L, Sládeček FX, Thorn S (2022). Understanding the drivers of β-diversity improves conservation prioritization for Central European dung beetles. Biol. Conserv..

[41] Cerasoli F, Fiasca B, Di Lorenzo T, Lombardi A, Tomassetti B, Lorenzi V, Vaccarelli I, Di Cicco M, Petitta M, Galassi DM (2023). Assessing spatial and temporal changes in diversity of copepod crustaceans: A key step for biodiversity conservation in groundwater-fed springs. Front. Environ. Sci..

[42] Cucchi F, Zini LL (2007). acque del Carso Classico. Mem. dell’Ist. Ital. di Speleol..

[43] Piccini L (2011). Speleogenesis in highly geodynamic contexts: The quaternary evolution of Monte Corchia multi-level karst system (Alpi Apuane, Italy). Geomorphology.

[44] Parise M, Benedetto L, Chieco M, Fiore A, Lacarbonara M, Liso IS, Masciopinto C, Pisano L, Riccio A, Vurro M, Bertrand C, Denimal S, Steinmann M, Renard P (2020). First outcomes of a project dedicated to monitoring groundwater resources in Apulia, southern Italy. Eurokarst 2018. Advances in the Hydrogeology of Karst and Carbonate Reservoirs.

[45] Santo A (1994). Idrogeologia dell’area carsica di Castelcivita (M. Alburni–SA). Geol. Appl. Idrogeol..

[46] Addesso R, Bellino A, D’Angeli IM, De Waele J, Miller AZ, Carbone C, Baldantoni D (2019). Vermiculations from karst caves: The case of Pertosa–Auletta system (Italy). Catena.

[47] Brancelj, A., Camacho, A. I., Fiers, F., Galassi, D., Gibert, J., Lefebure, T., Martin, P., Sket, B. & Valdecasas, A. G. Sampling manual for the assessment of regional groundwater biodiversity 1–110 (PASCALIS Project, V Framework Programme. Key Action 2: Global Change, Climate and Biodiversity. 2.2. 3 Assessing and Conserving Biodiversity, 2002).

[48] Cvetkov L (1968). Un filet phréatobiologique. Bull. Inst. Zool. Mus. Acad. Bulg. Sci..

[49] Borutzky, E.V. Fauna USSR. Crustacea, Volume III, No. 4. Freshwater Harpacticoida. 1–424 (Izdaniya Akademia Nauk, 1952). [Original in Russian but consulted for this work in the English translation by A. Mercado, published in 1964 by the Israel Program for Scientific Translation, Jerusalem].

[50] Dussart BH (1967). Les Copépodes des Eaux Continentales d'Europe Occidentale Tome I Calanoides et Harpacticoides 1–500.

[51] Dussart BH (1969). Les Copépodes des Eaux Continentales d'Europe Occidentale Tome II Cyclopoïdes et Biologie Quantitative.

[52] Boxshall GA, Halsey SH (2004). An Introduction to Copepod Diversity.

[53] Wells JBJ (2007). An annotated checklist and keys to the species of Copepoda Harpacticoida (Crustacea). Zootaxa.

[54] R Core Team. R: A language and environment for statistical computing. R Foundation for Statistical Computing, Vienna, Austria. Available at: https://www.R-project.org/ (Accessed 25 July 2023) (2022).

[55] Chao A (1984). Nonparametric estimation of the number of classes in a population. Scand. J. Stat..

[56] Burnham KP, Overton WS (1979). Robust estimation of population size when capture probabilities vary among animals. Ecology.

[57] Smith EP, van Belle G (1984). Nonparametric estimation of species richness. Biometrics.

[58] Okansen, J., Blanchet, F. G., Friendly, M., Kindt, R., Legendre, P., McGlinn, D., Minchin, P. R., O'Hara, R. B., Simpson, G. L., Solymos, P. & Stevens, M. H. Vegan: Community ecology package. R package version 2.6-2. Available at: https://CRAN.R-project.org/package=vegan (Accessed 25 July 2023) (2022).

[59] Colwell RK, Coddington JA (1994). Estimating terrestrial biodiversity through extrapolation. Philos. Trans. R. Soc. Lond. Ser. B Biol. Sci..

[60] Baselga A (2012). The relationship between species replacement, dissimilarity derived from nestedness, and nestedness. Glob. Ecol. Biogeogr..

[61] Baselga, A., Orme, D., Villeger, S., De Bortoli, J., Leprieur, F. & Logez, M. Betapart: Partitioning beta diversity into turnover and nestedness components. R package version 1.5.6. Available at: https://CRAN.R-project.org/package=betapart (Accessed 25th July 2023) (2022).

[62] Pebesma E (2018). Simple features for R: standardized support for spatial vector data. R J..

[63] Qian H, Ricklefs RE (2012). Disentangling the effects of geographic distance and environmental dissimilarity on global patterns of species turnover. Glob. Ecol. Biogeogr..

[64] Ferrier S, Manion G, Elith J, Richardson K (2007). Using generalized dissimilarity modelling to analyse and predict patterns of beta diversity in regional biodiversity assessment. Divers. Distrib..

[65] Mokany K, Ware C, Woolley SN, Ferrier S, Fitzpatrick MC (2022). A working guide to harnessing generalized dissimilarity modelling for biodiversity analysis and conservation assessment. Glob. Ecol. Biogeogr..

[66] Fitzpatrick, M., Mokany, K., Manion, G., Nieto-Lugilde, D. & Ferrier, S. GDM: Generalized Dissimilarity Modeling. R package version 1.5.0-9.1. Available at: https://CRAN.R-project.org/package=gdm (Accessed 25 July 2023) (2022).

[67] Fick SE, Hijmans RJ (2017). WorldClim 2: New 1-km spatial resolution climate surfaces for global land areas. Int. J. Climatol..

[68] Mammola S, Leroy B (2018). Applying species distribution models to caves and other subterranean habitats. Ecography.

[69] Naimi B, Hamm NA, Groen TA, Skidmore AK, Toxopeus AG (2014). Where is positional uncertainty a problem for species distribution modelling?. Ecography.

[70] Guisan A, Thuiller W, Zimmermann NE (2017). Habitat Suitability and Distribution Models: With Applications in R.

[71] Benjamini Y, Hochberg Y (1995). Controlling the false discovery rate: A practical and powerful approach to multiple testing. J. R. Stat. Soc. Ser. B (Methodol.).

[72] Zuur AF, Ieno EN, Elphick CS (2010). A protocol for data exploration to avoid common statistical problems. Methods Ecol. Evolut..

[73] Mammola S, Cardoso P, Culver DC, Deharveng L, Ferreira RL, Fišer C, Galassi DM, Griebler C, Halse S, Humphreys WF, Isaia M (2019). Scientists’ warning on the conservation of subterranean ecosystems. Bioscience.

[74] Stoch F, Galassi DMP (2010). Stygobiotic crustacean species richness: A question of numbers, a matter of scale. Hydrobiologia.

[75] Dole-Olivier MJ, Castellarini F, Coineau N, Galassi DM, Martin P, Mori N, Valdecasas A, Gibert J (2009). Towards an optimal sampling strategy to assess groundwater biodiversity: Comparison across six European regions. Freshw. Biol..

[76] Zagmajster M, Ferreira RD, Humphreys WF, Niemiller ML, Malard F, Malard F, Griebler C, Rétaux S (2023). Patterns and determinants of richness and composition of the groundwater fauna. Groundwater Ecology and Evolution.

[77] Soininen J, Heino J, Wang J (2018). A meta-analysis of nestedness and turnover components of beta diversity across organisms and ecosystems. Glob. Ecol. Biogeogr..

[78] Heino J, Alahuhta J, Fattorini S, Schmera D (2019). Predicting beta diversity of terrestrial and aquatic beetles using ecogeographical variables: Insights from the replacement and richness difference components. J. Biogeogr..

[79] Svenning J-C, Fløjgaard C, Baselga A (2011). Climate, history and neutrality as drivers of mammal beta diversity in Europe: Insights from multiscale deconstruction. J. Anim. Ecol..

[80] Thieltges DW, Hof C, Dehling DM, Brändle M, Brandl R, Poulin R (2011). Host diversity and latitude drive trematode diversity patterns in the European freshwater fauna. Glob. Ecol. Biogeogr..

[81] Viana DS, Figuerola J, Schwenk K, Manca M, Hobæk A, Mjelde M, Preston CD, Gornall RJ, Croft JM, King RA, Green AJ (2016). Assembly mechanisms determining high species turnover in aquatic communities over regional and continental scales. Ecography.

[82] Griffiths D (2017). Connectivity and vagility determine beta diversity and nestedness in North American and European freshwater fish. J. Biogeogr..

[83] Calligaris C, Mezga K, Slejko FF, Urbanc J, Zini L (2018). Groundwater characterization by means of conservative (δ18O and δ2H) and non-conservative (87Sr/86Sr) isotopic values: The classical Karst region aquifer case (Italy–Slovenia). Geosciences.

[84] Bruno MC, Cottarelli V, Grasso R, Latella L, Zaupa S, Spena MT (2018). Epikarst crustaceans from some Italian caves: Endemisms and spatial scales. Biogeogr. J. Integr. Biogeogr..

[85] Mohan C, Western AW, Wei Y, Saft M (2018). Predicting groundwater recharge for varying land cover and climate conditions—a global meta-study. Hydrol. Earth Syst. Sci..

[86] Sánchez-Fernández D, Rizzo V, Bourdeau C, Cieslak A, Comas J, Faille A, Fresneda J, Lleopart E, Millán A, Montes A, Pallares S (2018). The deep subterranean environment as a model system in ecological, biogeographical and evolutionary research. Subterr Biol..

[87] Mammola S, Piano E, Cardoso P, Vernon P, Domínguez-Villar D, Culver DC, Pipan T, Isaia M (2019). Climate change going deep: The effects of global climatic alterations on cave ecosystems. Anthropocene Rev..

[88] Di Cicco M, Di Lorenzo T, Fiasca B, Galmarini E, Vaccarelli I, Cerasoli F, Di Camillo AT, Galassi DM (2023). Some like it hot: Thermal preference of the groundwater amphipod *Niphargus longicaudatus* (Costa, 1851) and climate change implications. J. Therm. Biol..

[89] Ford DC, Williams PW (2007). Karst Hydrogeology and Geomorphology.

[90] Keil P, Schweiger O, Kühn I, Kunin WE, Kuussaari M, Settele J, Henle K, Brotons L, Peer G, Lengyel S, Moustakas A (2012). Patterns of beta diversity in Europe: The role of climate, land cover and distance across scales. J. Biogeogr..

[91] Eme D, Zagmajster M, Delić T, Fišer C, Flot JF, Konecny-Dupré L, Pálsson S, Stoch F, Zakšek V, Douady CJ, Malard F (2018). Do cryptic species matter in macroecology? Sequencing European groundwater crustaceans yields smaller ranges but does not challenge biodiversity determinants. Ecography.

