# Supplementary material for: Regional climate contributes more than geographic distance to beta diversity of copepods (Crustacea Copepoda) between caves of Italy
Source: Sci Rep. 2023 Dec 1;13:21243. doi: 10.1038/s41598-023-48440-7 (PMC10692170; doi:10.1038/s41598-023-48440-7)
Supplement: Supplementary file 1 — Supplementary Information. [file 41598_2023_48440_MOESM1_ESM.docx]

**Supplementary Materials**

**Regional climate contributes more than geographic distance to beta diversity of copepods (Crustacea Copepoda) between caves of Italy**

Emma Galmarini¹, Ilaria Vaccarelli¹^,^², Barbara Fiasca¹, Mattia Di Cicco¹, Mario Parise^3^, Isabella Serena Liso^3^, Leonardo Piccini^4^, Diana M. P. Galassi¹, Francesco Cerasoli¹*

¹ Department of Life, Health, and Environmental Sciences, University of L’Aquila, L’Aquila, Italy,

² University Institute of Higher Studies in Pavia, Pavia, Italy

^3^ Department of Earth and Environmental Sciences, University Aldo Moro, Bari, Italy

^4^ Department of Earth Science, University of Florence, Italy

* Correspondence: Francesco Cerasoli, francesco.cerasoli@univaq.it

**Table S1.** List of the recorded copepod species, along with the corresponding incidence data for each of the twelve sampled caves. * indicates a stygobitic species ; ° indicates a spot endemic species.

|  |  | Tr | Li | Co | GdV | TcU | Fi | St | Ca | Pe | GG | Ro | Pu |
| --- | --- | --- | --- | --- | --- | --- | --- | --- | --- | --- | --- | --- | --- |
| Copepoda Calanoida Sars G. O., 1903 |  |  |  |  |  |  |  |  |  |  |  |  |  |
| *Eudiaptomus intermedius* (Steuer, 1897) | *° | 0 | 0 | 0 | 0 | 0 | 1 | 0 | 0 | 0 | 0 | 0 | 0 |
| *Troglodiaptomus sketi* Petkovski, 1978 | * | 1 | 0 | 0 | 0 | 0 | 0 | 0 | 0 | 0 | 0 | 0 | 0 |
| Copepoda Cyclopoida Burmeister, 1835 |  |  |  |  |  |  |  |  |  |  |  |  |  |
| *Halicyclops dalmatinus* Petkovski, 1955 | * | 0 | 0 | 0 | 0 | 0 | 0 | 0 | 0 | 0 | 0 | 0 | 1 |
| *Eucyclops subterraneus intermedius* Damian, 1955 | * | 0 | 0 | 0 | 0 | 0 | 1 | 0 | 0 | 0 | 0 | 0 | 0 |
| *Eucyclops serrulatus* (Fischer, 1851) |  | 0 | 0 | 0 | 0 | 0 | 0 | 1 | 1 | 0 | 1 | 0 | 0 |
| *Eucyclops* sp.Ca1 | *° | 0 | 0 | 0 | 0 | 0 | 0 | 0 | 1 | 0 | 0 | 0 | 0 |
| *Paracyclops fimbriatus* (Fischer, 1853) |  | 0 | 0 | 0 | 0 | 0 | 0 | 0 | 0 | 0 | 0 | 0 | 0 |
| *Paracyclops imminutus* Kiefer, 1929 |  | 0 | 1 | 1 | 1 | 1 | 1 | 1 | 1 | 1 | 1 | 1 | 1 |
| *Acanthocyclops agamus* Kiefer, 1938 | * | 0 | 0 | 0 | 0 | 0 | 0 | 0 | 1 | 0 | 0 | 0 | 0 |
| *Acanthocyclops gr. venustus* |  | 0 | 0 | 0 | 0 | 0 | 1 | 0 | 0 | 0 | 0 | 0 | 0 |
| *Acanthocyclops hypogeus* (Kiefer, 1930) | *° | 0 | 1 | 0 | 0 | 0 | 0 | 0 | 0 | 0 | 0 | 0 | 0 |
| *Acanthocyclops robustus* (Sars G.O., 1863) |  | 1 | 0 | 0 | 0 | 0 | 1 | 1 | 0 | 0 | 0 | 0 | 0 |
| *Acanthocyclops vernalis* (Fischer, 1853) |  | 0 | 0 | 0 | 0 | 0 | 0 | 0 | 0 | 0 | 1 | 0 | 0 |
| *Macrocyclops albidus* (Jurine, 1820) |  | 1 | 0 | 0 | 0 | 0 | 1 | 0 | 0 | 0 | 0 | 0 | 0 |
| *Megacyclops viridis* (Jurine, 1820) |  | 1 | 0 | 0 | 0 | 0 | 0 | 1 | 1 | 1 | 0 | 0 | 0 |
| *Metacyclops stammeri* Kiefer, 1938 | * | 0 | 0 | 0 | 0 | 0 | 0 | 0 | 0 | 0 | 0 | 0 | 1 |
| *Diacyclops bicuspidatus bicuspidatus* (Claus, 1857) |  | 0 | 0 | 0 | 0 | 0 | 0 | 0 | 1 | 0 | 0 | 1 | 0 |
| *Diacyclops bicuspidatus lubbocki* (Brady, 1868) |  | 0 | 0 | 0 | 0 | 0 | 0 | 0 | 0 | 1 | 0 | 0 | 0 |
| *Diacyclops bisetosus* (Rehberg, 1880) |  | 0 | 0 | 1 | 0 | 0 | 0 | 0 | 0 | 0 | 0 | 1 | 0 |
| *Diacyclops charon* (Kiefer, 1931) | * | 1 | 0 | 0 | 0 | 0 | 0 | 0 | 0 | 0 | 0 | 0 | 0 |
| *Diacyclops clandestinus* (Yeatman, 1964) | * | 0 | 0 | 0 | 0 | 0 | 0 | 1 | 0 | 0 | 0 | 0 | 0 |
| *Diacyclops cosanus* Stella & Salvatori, 1954 | * | 0 | 0 | 0 | 0 | 0 | 1 | 0 | 0 | 0 | 0 | 0 | 0 |
| *Diacyclops languidoides* (Lilljeborg, 1901) |  | 0 | 0 | 0 | 0 | 0 | 0 | 0 | 0 | 1 | 1 | 0 | 0 |
| *Diacyclops lindae* Pesce, 1984 | * | 0 | 0 | 0 | 0 | 0 | 0 | 0 | 0 | 0 | 0 | 0 | 1 |
| *Diacyclops paolae* Pesce & Galassi, 1987 | * | 0 | 0 | 0 | 0 | 0 | 0 | 1 | 0 | 1 | 1 | 0 | 0 |
| *Diacyclops paralanguidoides* Pesce & Galassi, 1987 | * | 0 | 0 | 0 | 0 | 0 | 0 | 1 | 0 | 0 | 0 | 0 | 0 |
| *Diacyclops* sp.Pe1 | *° | 0 | 0 | 0 | 0 | 0 | 0 | 0 | 0 | 1 | 0 | 0 | 0 |
| *Diacyclops* sp.GG1 | *° | 0 | 0 | 0 | 0 | 0 | 0 | 0 | 0 | 0 | 1 | 0 | 0 |
| *Diacyclops* sp.GG/Sg1 | *° | 0 | 0 | 0 | 0 | 0 | 0 | 0 | 0 | 0 | 1 | 0 | 0 |
| *Diacyclops* sp.St1 | *° | 0 | 0 | 0 | 0 | 0 | 0 | 1 | 0 | 0 | 0 | 0 | 0 |
| *Diacyclops zschokkei* (Graeter, 1910) |  | 0 | 0 | 0 | 0 | 0 | 0 | 0 | 1 | 1 | 0 | 0 | 0 |
| *Speocyclops italicus* Kiefer, 1938 | * | 0 | 0 | 1 | 1 | 1 | 0 | 0 | 1 | 1 | 1 | 0 | 0 |
| *Speocyclops* sp.Fi1 | *° | 0 | 0 | 0 | 0 | 0 | 1 | 0 | 0 | 0 | 0 | 0 | 0 |
| *Stygocyclops teras* (Graeter, 1907) | * | 0 | 0 | 0 | 1 | 0 | 0 | 0 | 0 | 0 | 0 | 0 | 0 |
| *Hesperocyclops* sp.Ro1 | *° | 0 | 0 | 0 | 0 | 0 | 0 | 0 | 0 | 0 | 0 | 1 | 0 |
| Copepoda Harpacticoida Sars G. O., 1903 |  |  |  |  |  |  |  |  |  |  |  |  |  |
| *Pseudectinosoma kunzi* Galassi, 1997 | *° | 0 | 0 | 0 | 0 | 0 | 0 | 0 | 1 | 0 | 0 | 0 | 0 |
| *Pseudectinosoma* sp.Pu1 | *° | 0 | 0 | 0 | 0 | 0 | 0 | 0 | 0 | 0 | 0 | 0 | 1 |
| *Phyllognathopus viguieri* (Maupas, 1892) |  | 0 | 0 | 0 | 0 | 0 | 0 | 0 | 0 | 1 | 0 | 0 | 0 |
| *Nitocrella psammophila* Chappuis, 1954 | * | 0 | 0 | 0 | 0 | 0 | 1 | 0 | 0 | 0 | 0 | 0 | 0 |
| *Nitocrella stammeri* Chappuis, 1938 | * | 0 | 0 | 0 | 0 | 0 | 1 | 0 | 0 | 0 | 1 | 0 | 1 |
| *Nitocra hibernica* (Brady, 1880) |  | 0 | 0 | 0 | 0 | 0 | 0 | 0 | 1 | 1 | 0 | 0 | 0 |
| *Nitocra* sp.Pu1 | *° | 0 | 0 | 0 | 0 | 0 | 0 | 0 | 0 | 0 | 0 | 0 | 1 |
| *Bryocamptus zschokkei* (Schmeil, 1893) |  | 1 | 0 | 1 | 1 | 1 | 1 | 1 | 0 | 0 | 0 | 0 | 0 |
| *Bryocamptus pygmaeus* (Sars G.O., 1863) |  | 0 | 0 | 0 | 0 | 0 | 0 | 1 | 1 | 1 | 1 | 0 | 1 |
| *Bryocamptus* sp.Pe1 | *° | 0 | 0 | 0 | 0 | 0 | 0 | 0 | 0 | 1 | 0 | 0 | 0 |
| *Bryocamptus echinatus* (Mrázek, 1893) |  | 1 | 1 | 1 | 0 | 1 | 0 | 1 | 1 | 1 | 1 | 0 | 0 |
| *Canthocamptus staphylinus* (Jurine, 1820) |  | 0 | 0 | 0 | 0 | 0 | 0 | 1 | 0 | 0 | 0 | 0 | 0 |
| *Elaphoidella elaphoides* (Chappuis, 1924) | * | 0 | 1 | 0 | 0 | 0 | 0 | 0 | 0 | 0 | 1 | 1 | 0 |
| *Elaphoidella jeanneli* (Chappuis, 1928) | * | 1 | 0 | 0 | 0 | 0 | 0 | 0 | 0 | 0 | 0 | 0 | 0 |
| *Elaphoidella phreatica* (Chappuis, 1925) | * | 0 | 1 | 1 | 1 | 1 | 0 | 1 | 0 | 0 | 0 | 0 | 0 |
| *Elaphoidella plutonis plutonis* Chappuis, 1938 | * | 0 | 0 | 0 | 0 | 0 | 0 | 1 | 1 | 1 | 0 | 0 | 0 |
| *Elaphoidella plutonis quadrispinosa* Chappuis, 1938 | *° | 0 | 0 | 0 | 0 | 0 | 0 | 0 | 0 | 1 | 0 | 0 | 0 |
| *Elaphoidella* sp.GG1 | *° | 0 | 0 | 0 | 0 | 0 | 0 | 0 | 0 | 0 | 1 | 0 | 0 |
| *Epactophanes richardi* Mrázek, 1893 |  | 0 | 0 | 0 | 0 | 0 | 0 | 1 | 1 | 1 | 0 | 0 | 0 |
| *Hypocamptus brehmi* (Douwe, 1922) |  | 0 | 0 | 0 | 0 | 0 | 0 | 1 | 0 | 0 | 0 | 0 | 0 |
| *Maraenobiotus* sp.Fi1 | *° | 0 | 0 | 0 | 0 | 0 | 1 | 0 | 0 | 0 | 0 | 0 | 0 |
| *Moraria denticulata* Chappuis, 1938 | *° | 0 | 0 | 0 | 0 | 0 | 0 | 0 | 1 | 0 | 0 | 0 | 0 |
| *Moraria poppei meridionalis* Chappuis, 1929 |  | 0 | 0 | 0 | 0 | 0 | 1 | 0 | 1 | 0 | 1 | 0 | 0 |
| *Moraria* sp.Fi1 | *° | 0 | 0 | 0 | 0 | 0 | 1 | 0 | 0 | 0 | 0 | 0 | 0 |
| *Moraria* sp.Co1 | *° | 0 | 0 | 1 | 1 | 0 | 0 | 0 | 0 | 0 | 0 | 0 | 0 |
| *Moraria stankovitchi* Chappuis, 1924 | * | 0 | 0 | 0 | 0 | 0 | 1 | 0 | 0 | 0 | 0 | 0 | 0 |
| *Moraria varica* (Graeter, 1911) |  | 0 | 0 | 0 | 0 | 0 | 0 | 0 | 0 | 1 | 0 | 0 | 0 |
| *Pesceus schmeili* (Mrázek, 1893) |  | 0 | 0 | 0 | 0 | 0 | 0 | 1 | 0 | 1 | 0 | 0 | 0 |
| *Parastenocaris crenobia* Galassi, 1997 | * | 0 | 0 | 0 | 0 | 0 | 0 | 1 | 0 | 0 | 0 | 0 | 0 |
| *Parastenocaris reidae* Cottarelli, Bruno & Berera, 2007 | *° | 0 | 0 | 1 | 0 | 0 | 0 | 0 | 0 | 0 | 0 | 0 | 0 |
| *Parastenocaris* sp.GG1 | *° | 0 | 0 | 0 | 0 | 0 | 0 | 0 | 0 | 0 | 1 | 0 | 0 |
| *Parastenocaris* sp.Pe1 | *° | 0 | 0 | 0 | 0 | 0 | 0 | 0 | 0 | 1 | 0 | 0 | 0 |
| *Parastenocaris* sp.Pe2 | *° | 0 | 0 | 0 | 0 | 0 | 0 | 0 | 0 | 1 | 0 | 0 | 0 |
| *Stammericaris orcina* (Chappuis, 1938) | * | 0 | 0 | 0 | 0 | 0 | 0 | 0 | 1 | 1 | 0 | 1 | 0 |
| *Proserpinicaris proserpina* (Chappuis, 1938) | * | 0 | 0 | 0 | 0 | 0 | 0 | 0 | 0 | 1 | 0 | 0 | 0 |
|  |  |  |  |  |  |  |  |  |  |  |  |  |  |

**Table S2.** Extrapolated species richness values and corresponding standard errors (SE), across the 12 sampled caves, according to the Boostrap, Chao2, and first-order Jackknife (“Jackknife1”) non-parametric estimators. Obs/Est (%) = percent ratio between observed and estimated species richness. The last two columns show the percent ratio between observed species richness and the upper (Max.Est **=** estimated richness + SE) and lower (Min.Est = estimated richness - SE) extrapolated richness bounds, respectively.

| **Observed Richness** | **Richness estimator** | **Estimated Richness** | **Obs/Est (%)** | **SE** | **Obs/Max.Est (%)** | **Obs/Min.Est (%)** |
| --- | --- | --- | --- | --- | --- | --- |
| 71 | Bootstrap | 88.1 | 80.6 | 7.6 | 74.2 | 88.2 |
|  | Chao2 | 174.1 | 40.8 | 47.2 | 32.1 | 55.9 |
|  | Jackknife1 | 112.2 | 63.3 | 15.8 | 55.5 | 73.7 |

**Figure S1.** Species accumulation curves (shaded area: ± 2 × standard deviation) obtained using the Bootstrap, Chao2 and first-order Jackknife species richness estimators with 10000 sites’ permuting iterations. The horizontal green line corresponds to observed species richness (i.e., 71 copepod species) across the twelve sampled caves.
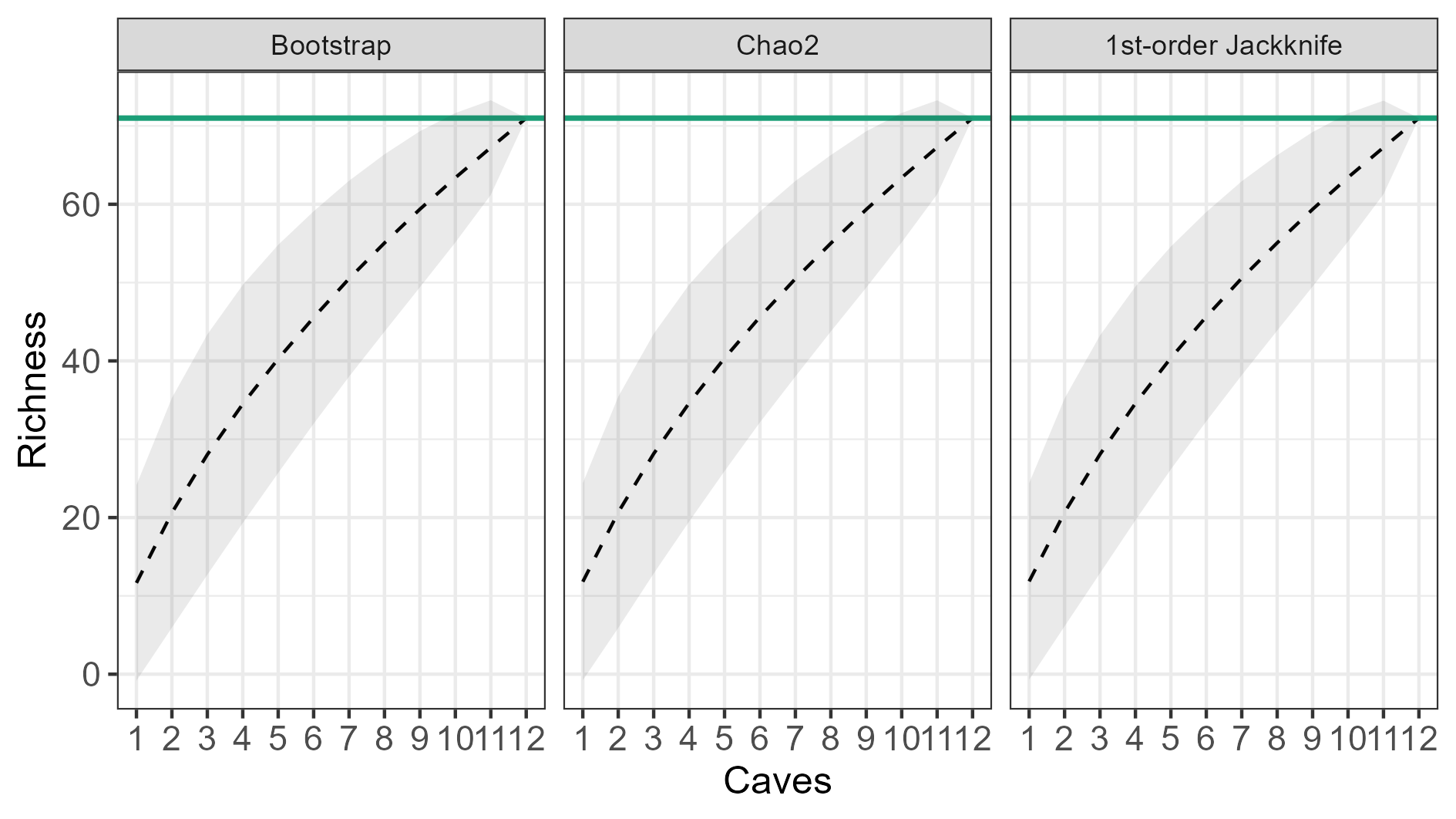


**Table S3.** Pairwise biotic dissimilarity for each pair of sampled caves, in terms of total taxonomic beta diversity and its two components (i.e., turnover and nestedness-resultant diversity).

|  | **Turnover** | | | | | | | | | | | |
| --- | --- | --- | --- | --- | --- | --- | --- | --- | --- | --- | --- | --- |
|  | Tr | Co | GG | Ro | Li | Pu | Fi | GdV | Ca | Pe | St | TcU |
| Tr | \ |  |  |  |  |  |  |  |  |  |  |  |
| Co | 0.75 | \ |  |  |  |  |  |  |  |  |  |  |
| GG | 0.78 | 0.62 | \ |  |  |  |  |  |  |  |  |  |
| Ro | 1 | 0.67 | 0.67 | \ |  |  |  |  |  |  |  |  |
| Li | 0.8 | 0.4 | 0.4 | 0.6 | \ |  |  |  |  |  |  |  |
| Pu | 1 | 0.88 | 0.62 | 0.83 | 0.8 | \ |  |  |  |  |  |  |
| Fi | 0.56 | 0.75 | 0.75 | 0.83 | 0.8 | 0.75 | \ |  |  |  |  |  |
| GdV | 0.83 | 0.17 | 0.67 | 0.83 | 0.6 | 0.83 | 0.67 | \ |  |  |  |  |
| Ca | 0.67 | 0.62 | 0.59 | 0.5 | 0.6 | 0.75 | 0.81 | 0.67 | \ |  |  |  |
| Pe | 0.67 | 0.62 | 0.59 | 0.67 | 0.6 | 0.75 | 0.88 | 0.67 | 0.39 | \ |  |  |
| St | 0.44 | 0.5 | 0.59 | 0.83 | 0.4 | 0.75 | 0.75 | 0.5 | 0.56 | 0.55 | \ |  |
| TcU | 0.6 | 0 | 0.4 | 0.8 | 0.4 | 0.8 | 0.6 | 0.2 | 0.4 | 0.4 | 0.2 | \ |
|  | **Nestedness** | | | | | | | | | | | |
|  | Tr | Co | GG | Ro | Li | Pu | Fi | GdV | Ca | Pe | St | TcU |
| Tr | \ |  |  |  |  |  |  |  |  |  |  |  |
| Co | 0.01 | \ |  |  |  |  |  |  |  |  |  |  |
| GG | 0.07 | 0.14 | \ |  |  |  |  |  |  |  |  |  |
| Ro | 0 | 0.05 | 0.16 | \ |  |  |  |  |  |  |  |  |
| Li | 0.06 | 0.14 | 0.33 | 0.04 | \ |  |  |  |  |  |  |  |
| Pu | 0 | 0 | 0.14 | 0.02 | 0.05 | \ |  |  |  |  |  |  |
| Fi | 0.12 | 0.08 | 0.01 | 0.08 | 0.1 | 0.08 | \ |  |  |  |  |  |
| GdV | 0.03 | 0.12 | 0.16 | 0 | 0.04 | 0.02 | 0.15 | \ |  |  |  |  |
| Ca | 0.11 | 0.14 | 0.01 | 0.25 | 0.23 | 0.1 | 0.01 | 0.17 | \ |  |  |  |
| Pe | 0.15 | 0.18 | 0.06 | 0.2 | 0.26 | 0.12 | 0.02 | 0.2 | 0.07 | \ |  |  |
| St | 0.21 | 0.21 | 0.03 | 0.09 | 0.36 | 0.11 | 0.03 | 0.27 | 0.02 | 0.03 | \ |  |
| TcU | 0.11 | 0.23 | 0.33 | 0.02 | 0 | 0.05 | 0.21 | 0.07 | 0.34 | 0.39 | 0.48 | \ |
|  | **Total** | | | | | | | | | | | |
|  | Tr | Co | GG | Ro | Li | Pu | Fi | GdV | Ca | Pe | St | TcU |
| Tr | \ |  |  |  |  |  |  |  |  |  |  |  |
| Co | 0.76 | \ |  |  |  |  |  |  |  |  |  |  |
| GG | 0.85 | 0.76 | \ |  |  |  |  |  |  |  |  |  |
| Ro | 1 | 0.71 | 0.83 | \ |  |  |  |  |  |  |  |  |
| Li | 0.86 | 0.54 | 0.73 | 0.64 | \ |  |  |  |  |  |  |  |
| Pu | 1 | 0.88 | 0.76 | 0.86 | 0.85 | \ |  |  |  |  |  |  |
| Fi | 0.68 | 0.83 | 0.76 | 0.91 | 0.9 | 0.83 | \ |  |  |  |  |  |
| GdV | 0.87 | 0.29 | 0.83 | 0.83 | 0.64 | 0.86 | 0.82 | \ |  |  |  |  |
| Ca | 0.78 | 0.77 | 0.6 | 0.75 | 0.83 | 0.85 | 0.82 | 0.83 | \ |  |  |  |
| Pe | 0.81 | 0.81 | 0.65 | 0.86 | 0.86 | 0.87 | 0.9 | 0.86 | 0.46 | \ |  |  |
| St | 0.66 | 0.71 | 0.62 | 0.92 | 0.76 | 0.86 | 0.78 | 0.77 | 0.58 | 0.58 | \ |  |
| TcU | 0.71 | 0.23 | 0.73 | 0.82 | 0.4 | 0.85 | 0.81 | 0.27 | 0.74 | 0.79 | 0.68 | \ |

**Table S4.** Pairwise geographic distance (in km) for each pair of sampled caves.

|  | Tr | Co | GG | Ro | Li | Pu | Fi | GdV | Ca | Pe | St | TcU |
| --- | --- | --- | --- | --- | --- | --- | --- | --- | --- | --- | --- | --- |
| Tr | \ |  |  |  |  |  |  |  |  |  |  |  |
| Co | 335.2 | \ |  |  |  |  |  |  |  |  |  |  |
| GG | 752.6 | 759.2 | \ |  |  |  |  |  |  |  |  |  |
| Ro | 605.0 | 673.6 | 176.3 | \ |  |  |  |  |  |  |  |  |
| Li | 15.2 | 329.8 | 765.2 | 618.7 | \ |  |  |  |  |  |  |  |
| Pu | 624.4 | 702.5 | 179.4 | 31.4 | 638.4 | \ |  |  |  |  |  |  |
| Fi | 262.7 | 226.4 | 561.4 | 454.9 | 268.4 | 482.5 | \ |  |  |  |  |  |
| GdV | 330.7 | 5.0 | 755.9 | 669.5 | 325.4 | 698.4 | 221.8 | \ |  |  |  |  |
| Ca | 585.5 | 563.2 | 196.1 | 177.7 | 596.7 | 207.1 | 371.2 | 560.0 | \ |  |  |  |
| Pe | 585.0 | 574.8 | 184.9 | 156.2 | 596.6 | 185.7 | 377.9 | 571.4 | 21.5 | \ |  |  |
| St | 381.2 | 329.3 | 433.2 | 348.1 | 389.5 | 378.2 | 135.4 | 325.6 | 239.3 | 248.3 | \ |  |
| TcU | 331.9 | 4.3 | 755.8 | 669.7 | 326.6 | 698.6 | 222.2 | 1.2 | 559.9 | 571.3 | 325.6 | \ |

**Table S5.** Results from the distance-decay models fitted for total between-cave beta diversity and its two components (i.e., turnover and nestedness-resultant diversity), using either a negative exponential or a power-law decay function. P-values ≤ 0.01 are highlighted in bold and indicate the models showing a statistically significant explanatory power based on the randomization algorithm implemented in the ‘decay.model’ function of the “beatapart” R package.

| **_Dissimilarity component_** | **_Model type_** | **_Intercept_** | **_Slope_** | **_pseudo-R_^2^** | **_p- value_** |
| --- | --- | --- | --- | --- | --- |
| _Turnover_ | _Negative exponential_ | _0.493_ | _0.001_ | _0.11_ | **_0.001_** |
| _Nestedness_ |  | _0.108_ | _0_ | _0.005_ | _0.528_ |
| _Total beta diversity_ |  | _0.576_ | _0.001_ | _0.234_ | **_< 0.001_** |
| _Turnover_ | _Power-law_ | _0.116_ | _0.159_ | _0.24_ | **_< 0.001_** |
| _Nestedness_ |  | _0.092_ | _0.006_ | _0.004_ | _0.587_ |
| _Total beta diversity_ |  | _0.175_ | _0.223_ | _0.429_ | **_< 0.001_** |

**Table S6.** Results from the one-tailed pairwise Wilcoxon Rank Sum Tests performed on the values of explained deviance and RMSE on test data, extracted from the Generalized Dissimilarity Models fitted for the three beta diversity metrics using as explanatory variables the between-cave geographic distance and the climatic averages computed over four distinct buffer radii (i.e., 0.5 km, 2.5 km, 5 km and 10 km). With respect to explained deviance, the alternative hypothesis for cell_ij_ of this Table was that median value from the GDMs fitted using the buffer indicated in the *i* row was significantly higher than the median value from GDMs fitted using the buffer indicated in the *j* column. The opposite alternative hypothesis (i.e., median value from the models fitted on the buffer indicated in the *i* row was lower than that of the models fitted on the buffer indicated in the *j* column) was set for RMSE. The p-values ≤ 0.01 are highlighted in bold.

|  | Buff_0.5 km | Buff_2.5 km | Buff_5 km | **Component** | **Evaluation metric** |
| --- | --- | --- | --- | --- | --- |
| Buff_2.5 km | 0.577 | \ | \ | Turnover | Explained deviance (%) |
| Buff_5 km | 0.132 | 0.121 | \ |  |  |
| Buff_10 km | **p < 0.001** | **p < 0.001** | **p < 0.001** |  |  |
| Buff_2.5 km | 1.000 | \ | \ | Nestedness |  |
| Buff_5 km | 1.000 | **p < 0.001** | \ |  |  |
| Buff_10 km | 1.000 | **p < 0.001** | 1.000 |  |  |
| Buff_2.5 km | 0.685 | \ | \ | Total beta div. |  |
| Buff_5 km | 0.685 | 0.685 | \ |  |  |
| Buff_10 km | **p < 0.001** | **p < 0.001** | **p < 0.001** |  |  |
| Buff_2.5 km | 0.604 | \ | \ | Turnover | RMSE_TestData |
| Buff_5 km | 0.099 | 0.076 | \ |  |  |
| Buff_10 km | **p < 0.001** | **p < 0.001** | **p < 0.001** |  |  |
| Buff_2.5 km | 0.555 | \ | \ | Total beta div. |  |
| Buff_5 km | 0.339 | 0.339 | \ |  |  |
| Buff_10 km | **p < 0.001** | **p < 0.001** | **p < 0.001** |  |  |

**Table S7.** Results from the one-tailed pairwise Wilcoxon Rank Sum Tests performed on the values of explained deviance extracted from the Generalized Dissimilarity Models fitted for turnover and total beta diversity using different sets of explanatory variables, with climatic averages computed over four distinct buffer radii (i.e., 0.5 km, 2.5 km, 5 km and 10 km). The tests were performed only for the combinations of dissimilarity component × sets of variables for which the preliminary Kruskal-Wallis Rank Sum Tests showed statistically significant differences among the models fitted on the distinct buffer radii.

“Prec”, “Temp” and “Dist” indicate precipitation-related variables, temperature-related variables and geographic distance, respectively. The alternative hypothesis for cell_ij_ of this Table was that median value from the GDMs fitted using the buffer indicated in the *i* row was significantly higher than the median from GDMs fitted using the buffer indicated in the *j* column. The p-values ≤ 0.01 are highlighted in bold.

|  | Buff_0.5 km | Buff_2.5 km | Buff_5 km | **Component** | **Variables** |
| --- | --- | --- | --- | --- | --- |
| Buff_2.5 km | 0.943 | \ | \ | Turnover | Dist alone |
| Buff_5 km | **p < 0.001** | **p < 0.001** | \ |  |  |
| Buff_10 km | **p < 0.001** | **p < 0.001** | **0.01** |  |  |
| Buff_2.5 km | 1.000 | \ | \ | Turnover | Prec alone |
| Buff_5 km | 1.000 | 1.000 | \ |  |  |
| Buff_10 km | 1.000 | 1.000 | 1.000 |  |  |
| Buff_2.5 km | 1.000 | \ | \ | Total beta div. |  |
| Buff_5 km | 1.000 | 1.000 | \ |  |  |
| Buff_10 km | 1.000 | 1.000 | 1.000 |  |  |
| Buff_2.5 km | 0.978 | \ | \ | Turnover | Temp alone |
| Buff_5 km | **p < 0.001** | **p < 0.001** | \ |  |  |
| Buff_10 km | **p < 0.001** | **p < 0.001** | **p < 0.001** |  |  |
| Buff_2.5 km | 0.961 | \ | \ | Total beta div. |  |
| Buff_5 km | 0.058 | **0.005** | \ |  |  |
| Buff_10 km | **p < 0.001** | **p < 0.001** | **p < 0.001** |  |  |
| Buff_2.5 km | 1.000 | \ | \ | Turnover | Dist+Prec |
| Buff_5 km | 1.000 | 1.000 | \ |  |  |
| Buff_10 km | 1.000 | 1.000 | 1.000 |  |  |
| Buff_2.5 km | 0.591 | \ | \ | Turnover | Dist+Temp |
| Buff_5 km | **p < 0.001** | **p < 0.001** | \ |  |  |
| Buff_10 km | **p < 0.001** | **p < 0.001** | **p < 0.001** |  |  |
| Buff_2.5 km | 0.740 | \ | \ | Total beta div. |  |
| Buff_5 km | 0.134 | 0.048 | \ |  |  |
| Buff_10 km | **p < 0.001** | **p < 0.001** | **p < 0.001** |  |  |
| Buff_2.5 km | 0.54 | \ | \ | Turnover | Prec+Temp |
| Buff_5 km | 0.889 | 0.889 | \ |  |  |
| Buff_10 km | **p < 0.001** | **p < 0.001** | **p < 0.001** |  |  |
| Buff_2.5 km | 0.888 | \ | \ | Total beta div. |  |
| Buff_5 km | 0.888 | 0.888 | \ |  |  |
| Buff_10 km | **p < 0.001** | **p < 0.001** | **p < 0.001** |  |  |

**Figure S2.** Scatterplots of observed versus predicted turnover values (i.e., β_sim_) within full-data GDMs fitted on climate averages computed over four distinct buffer radii around the locations of caves’ entrance: a) 0.5 km, (b) 2.5 km, (c) 5 km, (d) 10 km. The dashed red line indicates 1:1 ratio between observed and predicted values (i.e., “prefect” predictions), while the blue line represents a LOESS regression line summarizing the actual observed-predicted patterns. Spearman’s correlation coefficient between observed and predicted values is also shown on each plot.


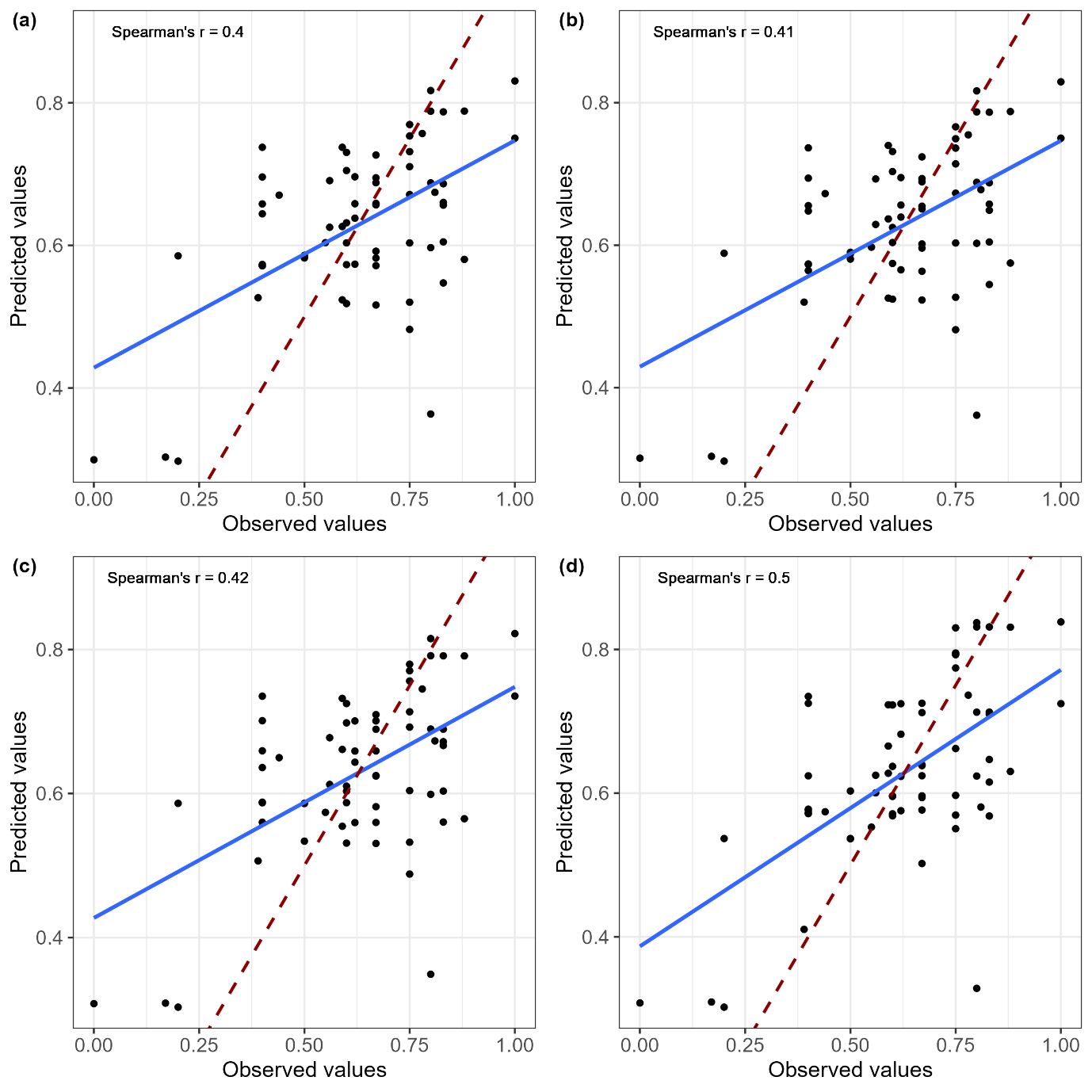


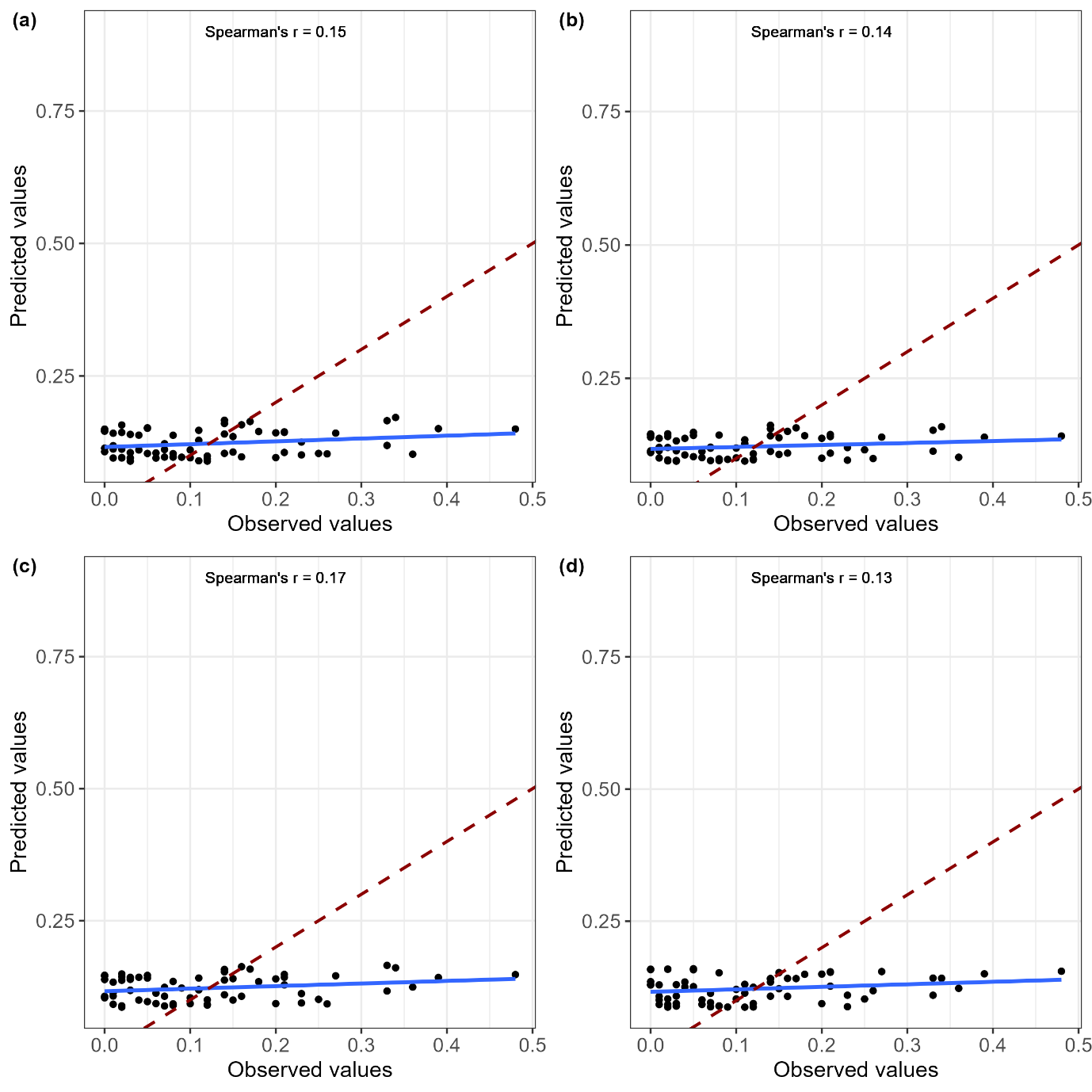
**Figure S3.** Scatterplots of observed versus predicted nestedness values (i.e., β_sne_) within full-data GDMs fitted on climate averages computed over four distinct buffer radii around the locations of caves’ entrance: a) 0.5 km, (b) 2.5 km, (c) 5 km, (d) 10 km. The dashed red line indicates a 1:1 ratio between observed and predicted values (i.e., “prefect” predictions), while the blue line represents a LOESS regression line summarizing the actual observed-predicted patterns. Spearman’s correlation coefficient between observed and predicted values is also shown on each plot.


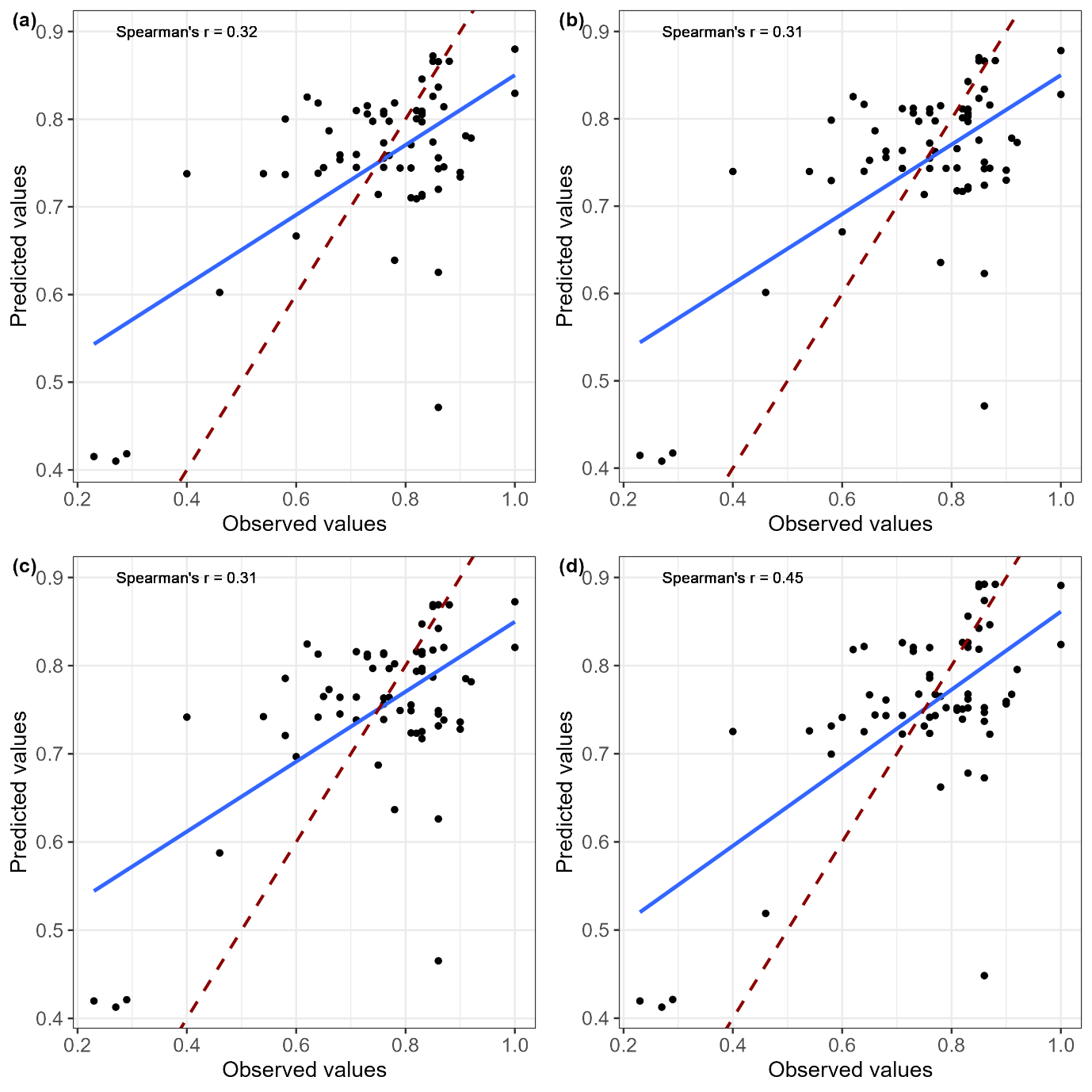
**Figure S4.** Scatterplots of observed versus predicted total beta diversity (i.e., β_sor_) within full-data GDMs fitted on climate averages computed over four distinct buffer radii around the locations of caves’ entrance: a) 0.5 km, (b) 2.5 km, (c) 5 km, (d) 10 km. The dashed red line indicates a 1:1 ratio between observed and predicted values (i.e., “prefect” predictions), while the blue line represents a LOESS regression line summarizing the actual observed-predicted patterns. Spearman’s correlation coefficient between observed and predicted values is also shown on each plot.


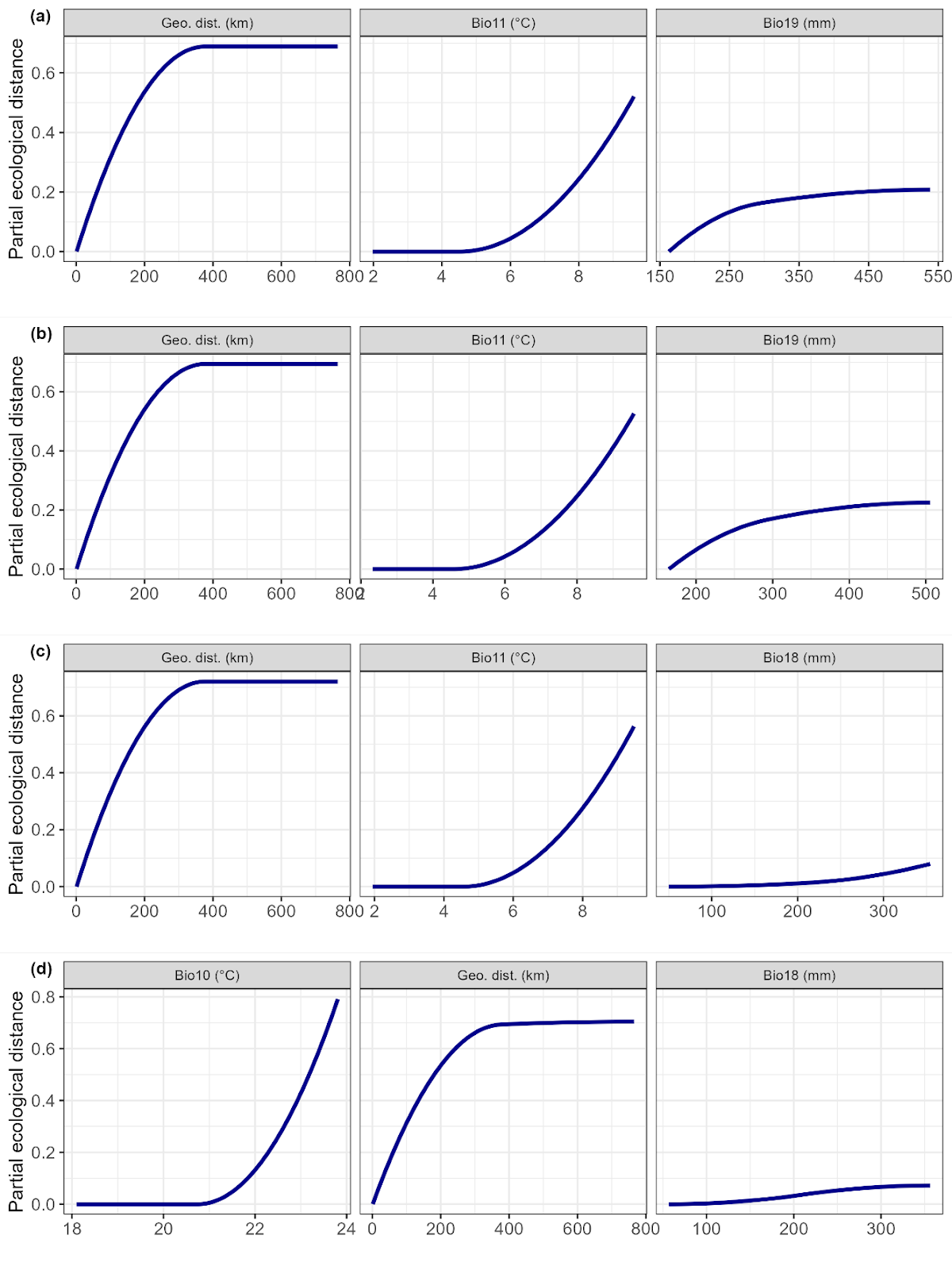
**Figure S5.** I-spline curves extracted from the full-data GDMs fitted with total beta diversity as response variable and including as explanatory variables climatic averages computed over a buffer around the caves’ entrance whose radius measured: (a) 0.5 km, (b) 2.5 km, (c) 5 km, and (d) 10 km. The curves show the relationship between modelled partial ecological distance and the values of the top-three variables in terms of relative contribution to the considered GDM. In each row, the order of the plots reflects the relative contribution of the variables, with the top contributing variable on the left.
